# High-Efficient Production of Adipose-Derived Stem Cell (ADSC) Secretome Through Maturation Process and Its Non-scarring Wound Healing Applications

**DOI:** 10.3389/fbioe.2021.681501

**Published:** 2021-06-16

**Authors:** Young-Hyeon An, Dae Hyun Kim, Eun Jung Lee, Dabin Lee, Mihn Jeong Park, Junghyeon Ko, Dong Wook Kim, Jiwan Koh, Hyun Sook Hong, Youngsook Son, Je-Yoel Cho, Ji-Ung Park, Sun-Dong Kim, Nathaniel S. Hwang

**Affiliations:** ^1^School of Chemical and Biological Engineering, Institute of Chemical Processes, Seoul National University, Seoul, South Korea; ^2^BioMax/N-Bio Institute, Seoul National University, Seoul, South Korea; ^3^Senior Science & Life, Inc., Seoul, South Korea; ^4^Department of Biochemistry, BK21 PLUS Program for Creative Veterinary Science Research and Research Institute for Veterinary Science, College of Veterinary Medicine, Seoul National University, Seoul, South Korea; ^5^Interdisciplinary Program in Bioengineering, Seoul National University, Seoul, South Korea; ^6^Department of Biomedical Science and Technology, Kyung Hee University, Seoul, South Korea; ^7^Department of Genetic Biotechnology and Graduate School of Biotechnology, Kyung Hee University, Yongin, South Korea; ^8^Department of Plastic and Reconstructive Surgery, Seoul National University Boramae Hospital, Seoul National University College of Medicine, Seoul, South Korea

**Keywords:** stem cells, secretome, proteomic analysis, skin regeneration, tissue repair

## Abstract

Recently, the stem cell-derived secretome, which is the set of proteins expressed by stem cells and secreted into the extracellular space, has been demonstrated as a critical contributor for tissue repair. In this study, we have produced two sets of high concentration secretomes from adipose-derived mesenchymal stem cells (ADSCs) that contain bovine serum or free of exogenous molecules. Through proteomic analysis, we elucidated that proteins related to extracellular matrix organization and growth factor-related proteins are highly secreted by ADSCs. Additionally, the application of ADSC secretome to full skin defect showed accelerated wound closure, enhanced angiogenic response, and complete regeneration of epithelial gaps. Furthermore, the ADSC secretome was capable of reducing scar formation. Finally, we show high-dose injection of ADSC secretome *via* intraperitoneal or transdermal delivery demonstrated no detectable pathological conditions in various tissues/organs, which supports the notion that ADSC secretome can be safely utilized for tissue repair and regeneration.

## Introduction

One of the promising techniques to treat severe non-healing wound is stem cell-based therapy, which reconstructs the wound healing process through trophic, paracrine, and immunomodulatory properties (Del Papa et al., 2015; Marfia et al., 2015). However, direct transplantation of stem cells is constrained by their low survival rate *in vivo* environment and potential contribution to teratoma formation (Lindeman and Visvader, 2010; Tu, 2010). To this end, the paracrine activity of the stem cells has attracted attention, suggesting that the use of the secreted molecules of stem cells would provide a therapeutic impact (Salgado et al., 2010; Lee et al., 2014; Robert et al., 2019). Secretome, also known as conditioned medium, is the complex set of the secreted molecules from stem cells, which includes extracellular vesicles and soluble fractions, such as cytokines and growth factors (Lee et al., 2010). Among the several sources of stem cells, enormous attention has been focusing on the adipose tissue-derived stem cells (ADSCs) due to the easy isolating procedure, liposuction, with a higher harvest rate than bone marrow (Hodgkinson et al., 2020). The ADSC-secretome has been proven to be useful not only for regenerating non-healing cutaneous and corneal wounds, cardiovascular disease, etc., but also to be utilized in a cosmeceutical application (Damous et al., 2018; Lombardi et al., 2019).

Skin wound healing is an orchestrated and highly regulated process, which includes inflammation, proliferation, matrix formation, and remodeling (Gurtner et al., 2008). When the process is disrupted, chronic non-healing wound occurs, resulting in not only significantly lowering the quality of life of the patient but also causing social costs due to the high prevalence rate (Jarbrink et al., 2016; Chan et al., 2017). In particular, the wound healing process is delayed or failed in severe conditions, such as diabetic ulcers and severe burns. Despite the increase of interest in secretome-based skin wound therapy, most studies are performed using serum-free secretome (SF) since the serum-containing secretome (SC) is susceptible to be contaminated (Shin et al., 2019); at the same time, the complicated procedure hinders the analysis of the SC (Weng et al., 2016). However, in the case of SF, the protein or cytokine level was highly reduced compared with SC; thus, a strategy is actually required to increase the amount of secreted molecules in the absence of serum proteins.

For these reasons, in this study, we produced a concentrated SF by culturing the ADSCs with the maturation process and subsequently evaporating it. It differs from previous methods that collect the secretome (or conditioned medium) at the time when the cell confluency is about 80–90% (Lombardi et al., 2019). In addition, we adjusted the level of both transforming growth factor-beta 1 (TGF-β1) and vascular endothelial growth factor (VEGF) in SF, which are representative growth factors for the wound healing process, to those of SC. Finally, the comparative study of the *in vivo* wound healing effects between SF and SC was carried out in mice dorsal skin wound model.

## Materials and Methods

### Adipose Tissue-Derived Stem Cells Isolation and Culture

Human adipose tissue-derived stem cells (ADSCs) were collected from five patients (age 32–60) with the liposuction process, which was implemented with consent from the patients and approval of the Boramae Medical Center (IRB No, 20160113/16-2016-3/021). After minced into small pieces, the adipose tissues were digested using 250 U/ml type I collagenase for 1 h, followed by neutralization with DMEM containing 10% fetal bovine serum (FBS). The digested mixture was filtered through a cell strainer of 40 μm. The suspension of cells was centrifuged, and the obtained cells were cultured in DMEM with 10% FBS.

### Preparation of SC and Serum-Free Secretome

The clinically graded secretome of ADSCs was produced with GMP-compliant manufacturing (Senior Lifescience, Co., Ltd.). ADSCs within passage 5 were cultured in 75 cm^2^ flasks using alpha-modified minimum essential medium (α-MEM), which was the absence of both phenol red and antibiotics. The medium was changed once ADSCs reached about 100% confluence, and the culture medium was continuously matured for 7 days to obtain both the SC and the SF without medium change. The secretome of each patient was collected and mixed homogeneously to minimize the donor variability.

### Enzyme-Linked Immunosorbent Assay

The level of both TGF-β1 and VEGF was quantified using enzyme-linked immunosorbent assay (ELISA) within the SF and SC, respectively. In the case of SF, the aqueous phase was dried out at room temperature using an evaporator for adjusting the protein levels similar to those of SC.

### Sample Preparation and Labeling for Proteomic Analysis

Only the SF was evaluated by proteomic analysis. A total of 240 μg protein of SF was subjected to filter-aided sample preparation (FASP) digestion (Sielaff et al., 2017). Proteins were trypsin digested at 37°C for 16 h, 3% trifluoroacetic acid was added into elute to stop the reaction, and the C18 tip column was used to remove salts. A total of 2 μg/5 μl of the digested peptide was used to analyze using liquid chromatography-mass spectrometry (LC-MS/MS).

### LC-MS/MS Analysis

Spectra raw data were acquired on an Orbitrap Fusion Lumos (Thermo Fisher Scientific, San Jose, CA) with EASY-nLC 1200 (Thermo Fisher Scientific, San Jose, CA). An autosampler was used to load 5 μl aliquots of the peptide solutions into an EASY column, Acclaim PepMap^TM^ 100 of i.d. 75 μm, length 2 cm, and particle size of 3 μm (Thermo Fisher Scientific, San Jose, CA). The trapped peptides were then separated on an EASY-Spray Column, C_18_ analytic-column of i.d. 75 μm and length 500 mm and 2 μm particle size (100 Å from Thermo Scientific). The mobile phases were composed of 100% water (A) and 100% acetonitrile (B), and each contained 0.1% formic acid. Liquid chromatography with 2 h gradient at a flow rate of 250 nl/min was used. During the chromatographic separation, the Orbitrap Fusion Lumos was operated in a data-dependent acquisition mode. Survey full scans were acquired on the mass range 400–1,600 *m*/*z*, maximum injection time of 100 ms, automatic gain control target 2 × 10^5^ ions with a resolution of 120,000, and analyzed using the Orbitrap. MS/MS precursors were selected from top *n* intense ions in 3 s between survey scans, which were fragmented by 37.5% higher-energy collisional dissociation. MS/MS was acquired on a maximum injection time of 54 ms, automatic gain control 5 × 10^4^ ions with a resolution of 30,000, and analyzed using the Orbitrap. Previously fragmented precursors were excluded for 30 s.

### Data Analysis for Protein Profiling

The raw data were processed with MaxQuant software (version 1.5.8.3) at default settings with unique peptide ≥ 2 and a minimum number of amino acid ≥ 6. Identified peaks were searched against a database of *Homo sapiens* from Uniprot^1^. Output files generated from Maxquant were subjected to Perseus (version 1.6.2.2) to perform bioinformatics analysis.

### *In vivo* Wound Healing Test

All the *in vivo* experimental procedures were approved by the IACUC of the Seoul National University (approval number: SNU-190916-2). The *in vivo* wound healing test was carried out using 8-week male balb/c-nude mice (OrientBio Co., Republic of Korea). Carbomer (Polygel CA, Happycall Co., Ltd., Republic of Korea) powder was added to both secretome solutions at 0.5% (*w*/*v*), followed by adding triethanolamine to prepare the gel formation by adjusting pH. For preparing the skin wound model, the dorsal skin of mice was pierced using a 6-mm diameter biopsy punch. The adhesive silicone chamber (CoverWell^TM^) was adhered to peripheral regions of the wound to avoid wound contraction and 30 μl of the SF- and SC-containing gel was applied, followed by covering the wound with Tegaderm^TM^ film dressing. At certain time points, the wound size was monitored and quantified compared with the initial wound size.

### Histological Analysis

At 7 and 14 days posttreatment of the wounds, the skin tissues were collected and fixed in 4% paraformaldehyde, subsequently processed to carry out the histological analysis. Hematoxylin and eosin (H&E) and Masson’s trichrome staining (MTC) were used to qualitatively compare the wound healing ability of both SF and SC.

### Panniculus Gap Measurement

Both the panniculus gap of adiposus and carnosus were analyzed at days 14 and 21 postwound, which are the gaps in length between the edge of the regenerated adipose and muscle layer, respectively. Based on the H&E images, these gaps were quantified using ImageJ (ImageJ Software).

### Epithelial Gap Measurement

The epithelial gap of the wounds was measured based on the immunohistochemical (IHC) staining of cytokeratin-10 (ab76318, Abcam) at day 14 postwound. The gap in length between the edges of cytokeratin-10-stained regions was measured using ImageJ.

### Measurement of Collagen Deposition, Extracellular Matrix Fiber Alignment, and Regeneration of Appendages

Collagen deposition, extracellular matrix (ECM) fiber alignment, and the number of skin appendages were measured based on the MTC staining images at day 21 postwound. For the collagen deposition, blue coloration was separated, which indicates the collagen, by thresholding of brightness, hue, and saturation in ImageJ. After which, the level of collagen deposition was analyzed by measuring the intensity and compared with each other normalized with the PBS group. ECM fiber alignment was quantified using the OrientationJ plugin available on ImageJ software, as described in a previous research (Chantre et al., 2018). Coherence values (dimensionless) were obtained from the region of interest in each sample. Lastly, the number of skin appendages was manually counted per image (×20 magnification) using at least 15 images.

### *In vivo* Vessel Formation

The *in vivo* angiogenic ability of each sample was evaluated by IHC staining of alpha-smooth muscle actin (α-SMA). The number of newly formed blood vessels was counted in a high-power field (HPF, ×40 magnification) images at least 15 images.

### Transdermal Delivery of the Secretome

The transdermal delivery of the secretome was tried using a combination of physical penetration enhancements, i.e., iontophoresis and a non-invasive metal roller. For visualizing the delivered components, we conjugated the near-infrared dye, ZW800-1C-NHS ester, to SF by reacting 2 h at room temperature, followed by filtration against a 7-kDa MWCO centrifugal membrane tube. The ZW800-1C-conjugated SF was applied to *ex vivo* porcine skin and treated with physical enhancements. After 15 min and 1 h, the sample was wiped out, and skin surface was washed with saline, and the skin was imaged using the fluorescence-assisted resection and exploration (FLARE) system with 3.6 mW/cm^2^ of 750 nm excitation light and white light (400–650 nm) at 5,500 lux. The semiquantitative analysis was carried out by measuring the integrated density of the fluorescent images using Image J (Image J Software).

### Pathological Analysis of the Secretome via Intraperitoneal Injection and Skin Application

Intraperitoneal injection (IP) was carried out by injecting 50 μl of SF into the 8-week female balb-c mice (OrientBio Co.) every 2 days to estimate the apoptotic response of the organs. After 7 and 14 days postinjection, the mice were euthanized, and the heart, liver, spleen, lung, and kidney were collected. The SF was also applied to the dorsal skin every 2 days for 14 days. The collected tissues were processed to implement the H&E and TUNEL staining.

### Statistical Analysis

Statistical significance was determined by one-way analysis of variance (ANOVA) by Tukey’s multiple comparisons method with GraphPad Prism 9 Software (Graphpad Software, San Diego, CA, United States). All data are presented as mean ± standard deviation (SD).

## Results

### Preparation of the Serum-Free Secretome and Serum-Containing Secretome

**SCHEME 1:**
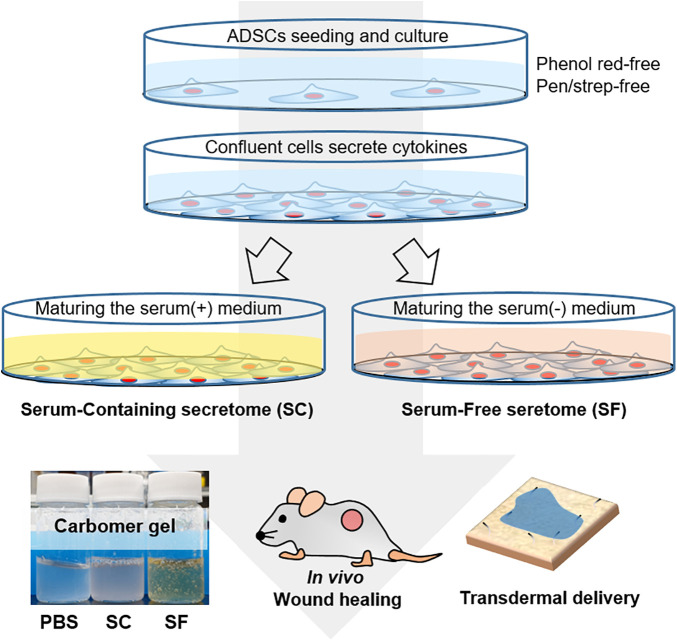
The schematic illustration of the experimental procedure. The serum-containing (SC) and serum-free secretome (SF) were harvested by maturing the fully confluent adipose tissue-derived stem cells (ADSCs). The secretome was applied to the *in vivo* skin wound and tried to be delivered transdermally using the *ex vivo* porcine skin.

The SF and SC were prepared using the primary human ADSCs from independent donors. We harvested the secretome by maturing ADSCs, which could maximize the secretion efficacy of the cytokines, growth factor, and protein molecules, according to the workflow in Scheme 1. Since both TGF-β1 and VEGF are essential in the wound healing process, we analyzed these two factors to comparatively evaluate the *in vivo* wound healing effects. Initially, the SC exhibited the level of TGF-β1 and VEGF of about 1.5 and 19.5 ng/ml, respectively. While the original levels of the proteins in SF were far lower than those of the SC; however, we could concentrate the SF through a drying out method (Table 1).

**TABLE 1:** The concentration of growth factors in ADSC-derived secretome (ng/ml).

Samples	TGF-β1	VEGF
Serum-free secretome (SF)	1.5	24.9
Serum-containing secretome (SC)	1.5	19.5

### Proteomic Analysis of the Serum-Free Secretome Reveals the Biological Process Highly Involved in the Wound Healing Process

**FIGURE 1:**
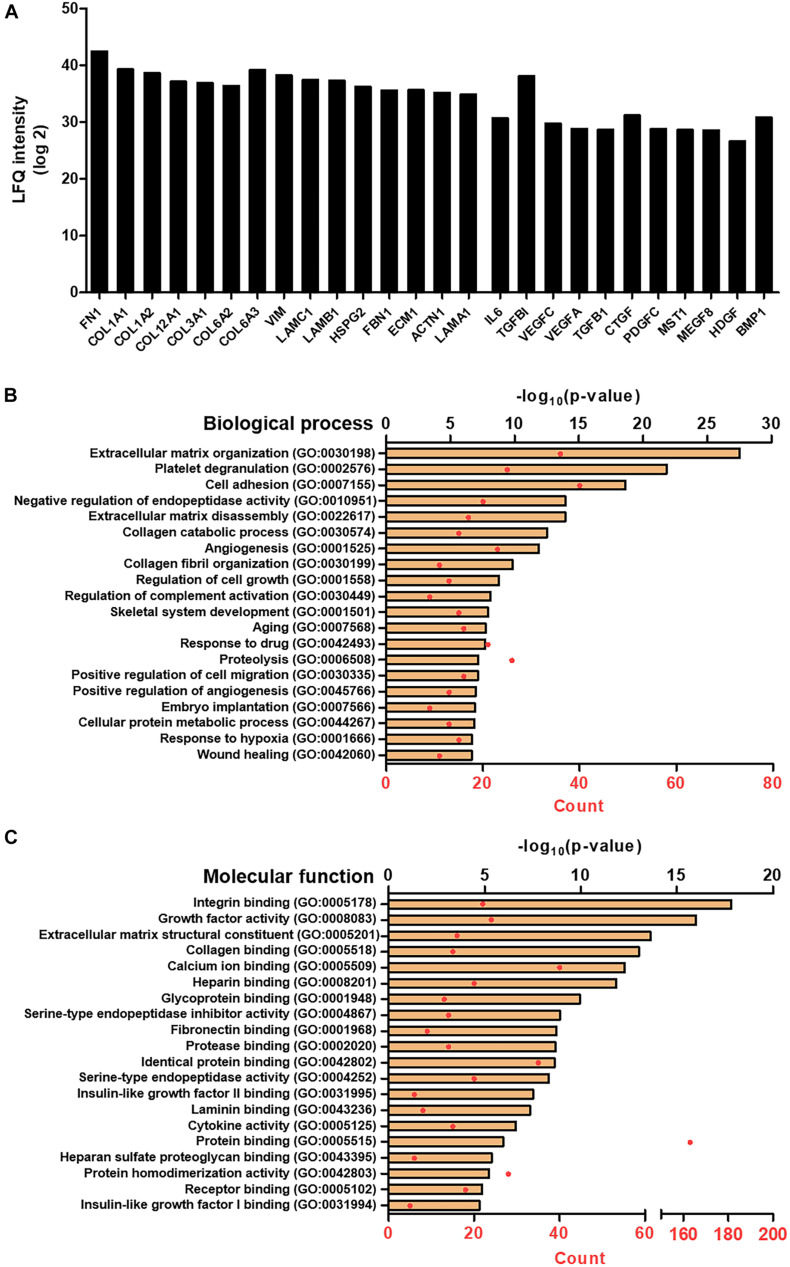
Proteomic analysis of SF. **(A)** LFQ intensity of representative extracellular matrix protein and growth factors, measured by LC/MS-MS. Gene ontology (GO) analysis representing the top 20 GO terms and its number of enrichment in **(B)** biological process and **(C)** molecular function.

The proteomic analysis of the SC revealed that a total of 704 different proteins were consist of the secretomes in the absence of the serum proteins. Among them, the representative 20 proteins of the extracellular matrix and growth factors are shown in Figure 1A. The proteins that are involved in the matrix organization, e.g., fibronectin, collagen families, and vimentin, were mostly identified with strong signals, and a variety of growth factors, such as the VEGF family, TGF-β1, and connective tissue growth factor (CTGF), were observed. The protein name, according to the abbreviation, is described in Table 2. In addition, by combining the proteomics with gene ontology (GO) analysis, we could comprehensively classify the secreted proteins on the basis of both the biological process and molecular function (Figures 1B,C). The top 20 of the biological process of SF revealed that it would be highly involved in the wound healing process, exhibiting the GO terms of an extracellular matrix organization, angiogenesis, cell migration, wound healing, etc.

**TABLE 2:** The representative extracellular matrix proteins and growth factors among the identified secreted molecules.

	Protein name	Abbreviation
Extracellular matrix proteins	*Fibronectin; Anastellin; Ugl-Y1; Ugl-Y2; Ugl-Y3*	*FN1*
	*Collagen alpha-1 (I) chain*	*COL1A1*
	*Collagen alpha-3 (VI) chain*	*COL6A3*
	*Collagen alpha-2 (I) chain*	*COL1A2*
	*Vimentin*	*VIM*
	*Laminin subunit gamma-1*	*LAMC1*
	*Laminin subunit beta-1*	*LAMB1*
	*Collagen alpha-1 (XII) chain*	*COL12A1*
	*Collagen alpha-1 (III) chain*	*COL3A1*
	*Collagen alpha-2 (VI) chain*	*COL6A2*
	*Basement membrane-specific heparan sulfate proteoglycan core protein; Endorepellin; LG3 peptide*	HSPG2
	*Fibrillin-1*	*FBN1*
	*Extracellular matrix protein 1*	*ECM1*
	*Alpha-actinin-1*	*ACTN1*
	*Laminin subunit alpha-1*	*LAMA1*
Growth factors	*Interleukin-6*	*IL6*
	*Transforming growth factor-beta-induced protein ig-h3*	*TGFBI*
	*Vascular endothelial growth factor C*	*VEGFC*
	*Vascular endothelial growth factor A*	*VEGFA*
	*Transforming growth factor beta-1; Latency-associated peptide*	*TGFB1*
	*Connective tissue growth factor*	*CTGF*
	*Platelet-derived growth factor C; Platelet-derived growth factor C, latent form; Platelet-derived growth factor C, receptor-binding form*	*PDGFC*
	*Hepatocyte growth factor-like protein; Hepatocyte growth factor-like protein alpha chain; Hepatocyte growth factor-like protein beta chain*	*MST1*
	*Multiple epidermal growth factor-like domains protein 8*	*MEGF8*
	*Hepatoma-derived growth factor*	*HDGF*

### The Serum-Containing Secretome Leads to Faster Wound Closure Than the Serum-Free Secretome

**FIGURE 2:**
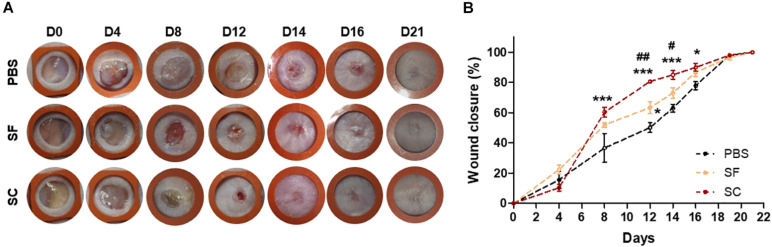
The ADSC secretome accelerated wound closure. **(A)** Photographs of the wound (rubber ring diameter = 9 mm). **(B)** Wound closure profiles by measuring the wound size (*compared with the PBS group; ^#^compared with the SF group;*^#^*p* < 0.05; ^##^*p* < 0.01; ***p < 0.001).

We compared the wound closure rate between SF and SC with tuning the level of TGF-β1 and VEGF through the drying out process. When applying the SF and SC to the mice skin wound every 2 days, the wound healing rate was significantly faster than that of the PBS-applied group (Figure 2). Although the SF contained a similar level of TGF-β1 and even the higher VEGF contents, the SC exhibited a much effective wound closure ability than the SF.

**FIGURE 3:**
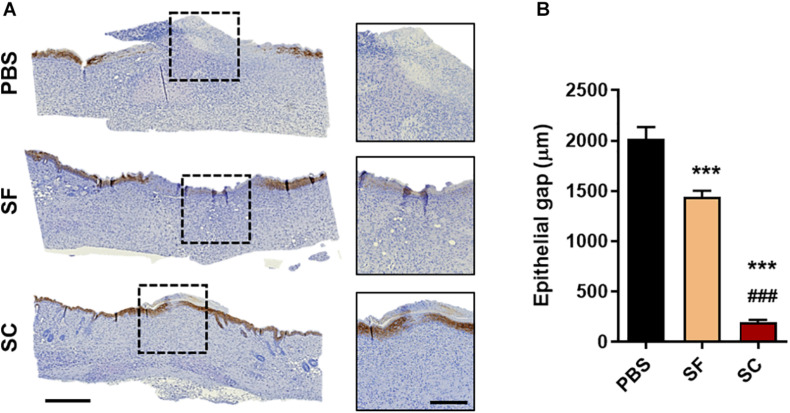
Immunohistochemical staining of cytokeratin-10 on day 14. **(A)** Representative microscopy images (Scale bar = 500 μm in low magnified images and 200 μm in high magnified images) and **(B)** the gap between the regenerated epithelial cells (***^###^*p* < 0.001).

**FIGURE 4:**
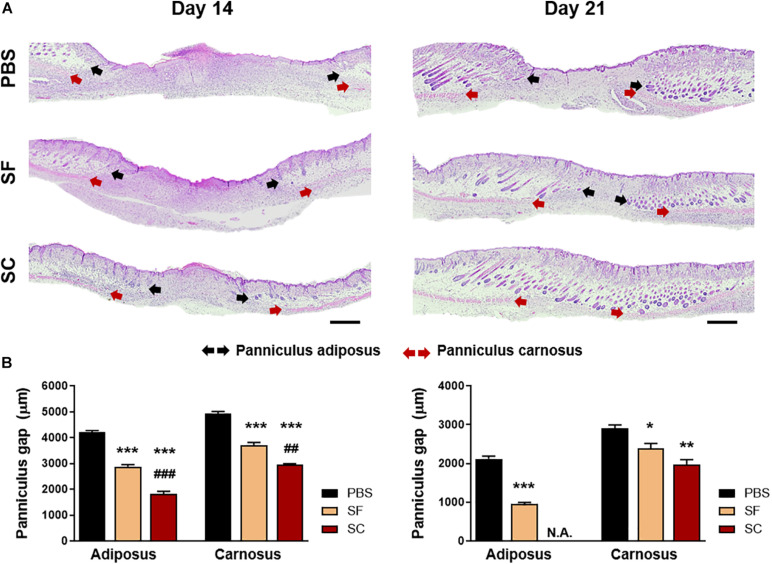
Hematoxylin and eosin (H&E) staining of the wound on days 14 and 21. **(A)** Light microscopy images of the wound bed representing the panniculus adiposus (black arrow) and carnosus (red arrow) (Scale bar = 500 μm). **(B)** Quantitative measurement of the panniculus gap at days 14 and 21 (*compared with the PBS group; ^#^compared with the SF group; *p < 0.05; **^##^*p* < 0.01; ***^###^*p* < 0.001).

The result of the IHC staining of cytokeratin-10 supported the faster wound closure of the SC, which revealed the narrow epithelial gap (Figure 3). In the case of SF, the epithelial gap was significantly reduced compared with the PBS group; however, it did not show as much effect as SC. In addition, the H&E staining showed that the SC provided outstanding wound healing effects not only on the superficial wound closure but also on the regeneration of internal skin tissue (Figure 4). The adipose (adiposus) and muscle (carnosus) layers were regenerating in the wound bed, narrowing those gaps between the edges of native tissue. The SC showed the narrowest gaps of both layers at day 14 postwound; even the adiposus layer in SC was clearly regenerated. As a result, the SC has a superior wound healing ability than SF with showing faster wound closure and internal tissue regeneration.

### The Serum-Containing Secretome Enhances Tissue Remodeling Compared With the Serum-Free Secretome

**FIGURE 5:**
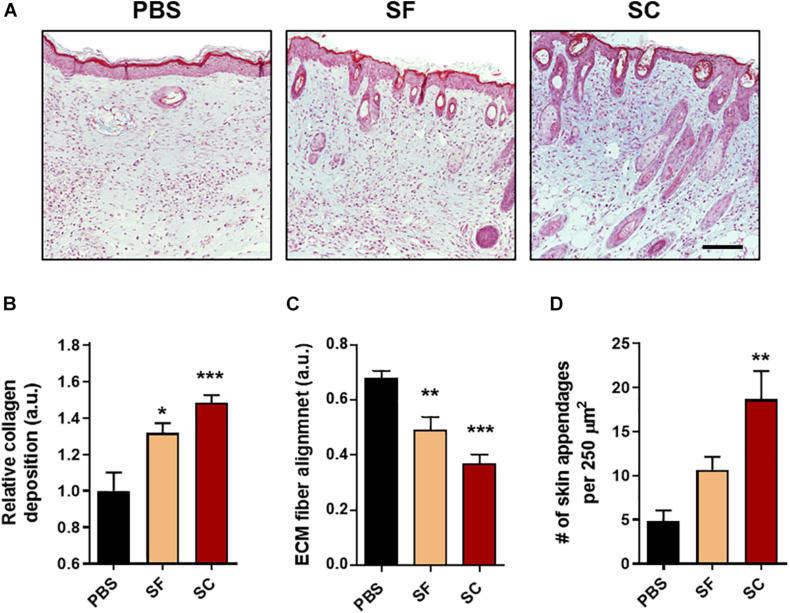
Masson’s trichrome (MTC) staining of the wounds at day 21. **(A)** Representative light microscopy images (Scale bar = 100 μm). The qualitative analysis of the skin regeneration **(B)** collagen deposition, **(C)** extracellular matrix (ECM) fiber alignment, and **(D)** the number of skin appendages (*compared with the PBS group; ^#^compared with the SF group; ^∗^*p* < 0.05; **p < 0.01; ***p < 0.001).

After the proliferative phase during the wound healing process, the tissue granulation and remodeling process begin, and these are determined by the collagen synthesis and ECM remodeling (Gurtner et al., 2008). At 21 days postwound, the MTC showed that the collagen synthesis of the SC groups was highly increased in the wound bed compared with both the SC and PBS groups (Figure 5). Moreover, the alignment of ECM fiber in the SC group revealed the lowest values, which indicates the wound bed became most similar to that of the native tissue (Ferguson and O’Kane, 2004; Chantre et al., 2018). Remarkably, the number of newly formed skin appendages was significantly higher in the SC group than that of the SF group.

**FIGURE 6:**
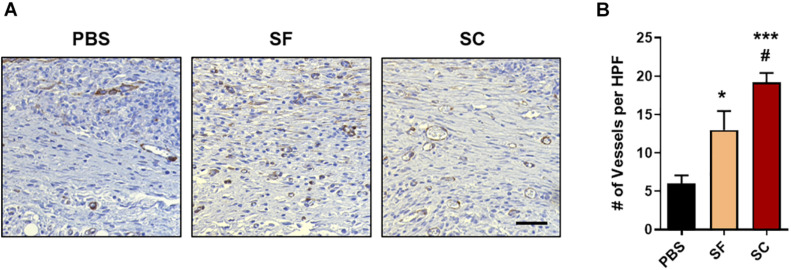
*In vivo* angiogenesis evaluation with the immunohistochemical staining (IHC) of alpha-smooth muscle actin (α-SMA). **(A)** Representative light microscopy images at regenerated wounds (Scale bar = 100 μm). **(B)** The number of newly formed vessels in the high-power field (HPF) magnification images (*n* = 10–15) (*compared with the PBS group; ^#^compared with the SF group; ^*,#^*p* < 0.05; ***p < 0.001).

Angiogenesis is one of the other criteria confirming tissue remodeling during the wound healing process (Tonnesen et al., 2000). The IHC staining of α-SMA indicated that SC induced the largest number of angiogenesis, and the SF also showed the angiogenic ability but inferior to the SC (Figure 6). Also, the vasculature in the SC group was enlarged than that of the SF group. As a result, it was proved that the SC showed increasing collagen synthesis, the formation of skin appendages, and promoted angiogenic response, that is, the SC enhances the tissue remodeling process compared with the SF.

### Intraperitoneal Injection and Pathological Analysis

**FIGURE 7:**
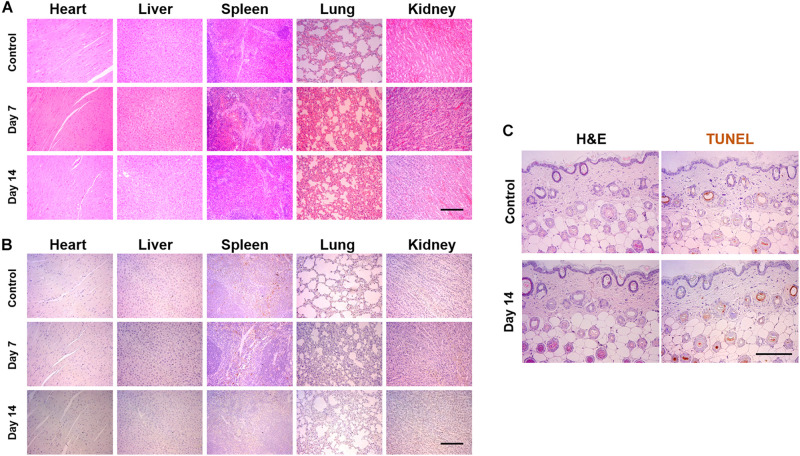
Pathological analysis of SF *via* intraperitoneal injection and applying to the skin surface. The 50 μl of SF was injected every other day until 2 weeks or applied to the dorsal skin surface of mice. **(A)** Hematoxyline and eosin (H&E) and **(B)** TUNEL staining of the tissues harvested from the heart, the liver, the spleen, the lung, and the kidney. **(C)** H&E- and TUNEL-stained images of the skin, which did not display any toxical responses (Scale bar = 200 μm).

To confirm the biosafety of the SF, we injected SF into the peritoneal region in mice and carried out the pathological analysis using H&E and TUNEL staining. After 7 and 14 days postinjection, the tissues were harvested from the heart, the liver, the spleen, the lung, and the kidney. H&E staining demonstrated that the tissues of SF-injected mice showed similar microscopic morphology compared with those of healthy mice (control), and abnormal changes were not observed in the tissues (Figure 7A). Moreover, to confirm the apoptosis level in the normal tissues, the TUNEL assay was also performed against the heart, the liver, the spleen, the lung, and the kidney (Figure 7B). Similar to the healthy group, there were few apoptotic cells in the harvest tissues of the SF-treated group, both on days 7 and 14; however, the biosafety of the secretome should be further investigated in a dose-dependent manner.

### Transdermal Delivery of Secretome Using Non-invasive Physical Penetration Enhancers

**FIGURE 8:**
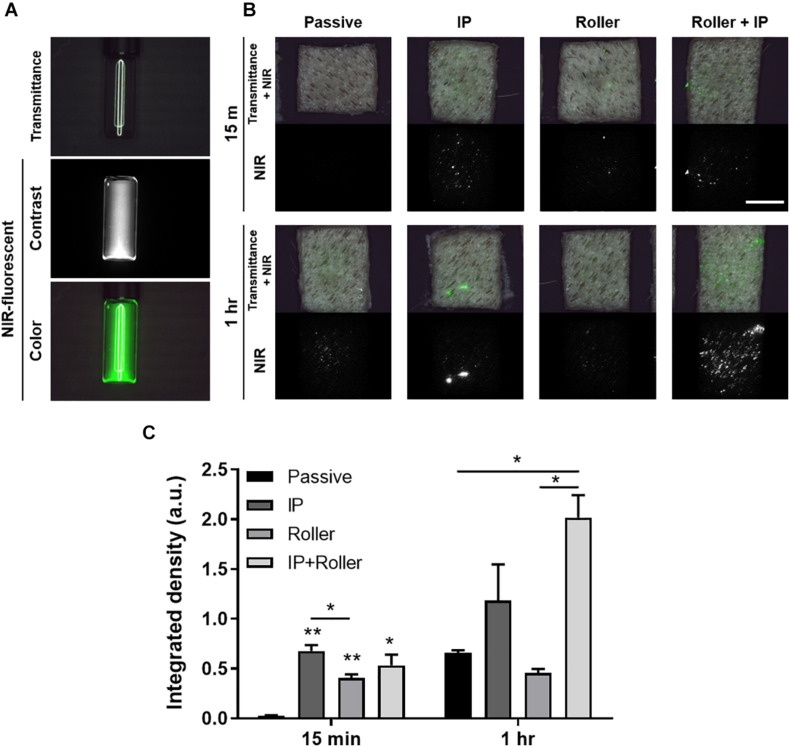
*Ex vivo* transdermal delivery of NIR dye-labeled SF. **(A)** The NIR dye, ZW800-1C-NHS-ester, was conjugated with proteins in the secretome. **(B)** Macroscopic visualization of transport of secretome into the *ex vivo* porcine skin with the assistance of physical penetration enhancers (PPEs). **(C)** The quantification data based on the FLARE images indicated the combinatorial application of IP and roller enhanced the transport of secretome transdermally (*p < 0.05; **p < 0.01) (Scale bar = 1 cm).

We finally tried to deliver the molecules in the secretome through transdermally using the physical penetration enhancers (PPEs), i.e., iontophoresis and non-invasive metal roller. For visualization, we labeled the secretome with NHS ester-conjugated ZW800-1C, near-infrared (NIR) fluorescent dye (Figure 8A). This NIR-labeled secretome was applied to the *ex vivo* porcine skin, and PPEs were exerted on the skin, after 15 min and 1 h, then the skin tissues using the FLARE system (Figure 8B), followed by implementing the semiquantitative analysis (Figure 8C). A little portion of the secretome was absorbed into the skin in the passive and roller groups, where the secretome was nearby the follicular regions. However, in the case of the iontophoresis (IP) group, the fluorescent signals appeared in several regions, even in other than follicular regions. In addition, the most abundant secretome was absorbed into the skin when the combination of roller and IP was applied during 1 h. As a result, it was confirmed that the transdermal delivery of the secretome was significantly increased in the usage of additional stimulation, whereas hardly achieved by passive diffusion alone.

## Discussion

The most crucial finding in this study is that we have developed a maturing method that can maximize the productive efficiency of the stem cell secretome. In this study, we produced the ADSC secretome with a GMP-compliant process, which could lower the possible contamination. It contained a remarkably high amount of protein molecules for wound healing, i.e., TGF-β1 and VEGF. We could obtain the SF and concentrated it in ambient conditions. This allowed the SF to have a similar level of proteins to that of SC, and their *in vivo* wound healing ability was evaluated.

The secreted substances from stem cells, such as exosome and secretome, possess the cellular information to exert biological activity. As a cell-free therapy, they have been extensively studied not only for wound treatment but also administered through various routes for disease treatment (Beer et al., 2016; Konala et al., 2016; Guan et al., 2017; Laggner et al., 2020). In the case of secretome, both quality control and analysis are the most significant barriers to their clinical usage. Although recent advances in analytical techniques make it feasible to determine the protein molecules, the serum-containing secretome is susceptible to contamination, which interrupts mass production, storage, distribution, and commercialization (Shin et al., 2019). While the serum-free secretome is relatively less susceptible to contamination and has a diminished immune response, the amount of secreted molecules is significantly reduced. Some strategies, such as freeze-drying and supercritical fluid, have been applied as a drying method to store the secretome and elevate the concentration of proteins (Bari et al., 2018); however, this procedure may also hinder the stability of the secretome proteins (Bari et al., 2018).

The conventional method of harvesting a secretome (or conditioned medium) proceeds in cell confluency about 70–90% up to 48 h (Kalinina et al., 2015; Park et al., 2018). In contrast, we used the maturation culturing method by continuously culturing the ADCSs from a fully confluent state and harvested the medium after 7 days, which could be applicable to obtain the SF. To compare the yield of secretome, we chose two representative growth factors, i.e., TGF-β1 and VEGF, since they exert crucial roles in the wound healing process. In previous studies, VEGF contents in stem cell secretome (or conditioned medium) were at most under 0.5 ng/ml (Zisa et al., 2009; Ge et al., 2018). Interestingly, SC, in this study, represented the concentration of about 20 ng/ml without any additional processes (Table 1), which is extraordinarily superior to other works. For TGF-β1, we could harvest about 1.5 ng/ml in pristine SC, but there were few studies for comparison. Above all, in the proteomic analysis of SF (Figure 1), we confirmed that there were lots of proteins relevant to an ECM organization, e.g., fibronectin, collagen subunits, vimentin, etc. (Table 2). It suggests that the maturing culture of ADSCs promotes the secretion of ECM proteins as well as maximizes the productive efficacy of the secretome.

An apparent reason for obtaining a high level of secreted proteins is that the absolute amount of cells in this study (100% confluent) was higher than that of the conventional method (70–90% confluent)—the crucial point of this study to optimize the maturing conditions where apoptosis did not occur. Besides, we considered that the maturing process-induced hypoxia might be the main reason for accelerating the secretion of a high amount of VEGF since the level of VEGF expression increased in hypoxia conditions (Chang et al., 2013). Moreover, some works demonstrated that secretomes from apoptotic cells had elevated levels of proangiogenic factors and also an impact on tissue regeneration and anti-inflammatory functions (Beer et al., 2015; Simader et al., 2019; Alegre, 2020; Medina et al., 2020). We hypothesize that ADSCs in the maturing process stress-related signaling which may have enhanced the secretion of the biological molecules that eventually facilitated the tissue repair process.

Wound healing is a complex and orchestrated process that several processes are integrated and overlapped. For this reason, secretome-based therapy is beneficial to treat the wound as it contains anti-inflammatory factors, promotes cell mitogenesis, and induces neovascularization (Estrada et al., 2009; Mildner et al., 2013; Lombardi et al., 2019). We found out that both SF and SC promoted overall wound healing, particularly wound closure (Figure 2), re-epithelization (Figure 3), proliferation (Figure 4), tissue remodeling (Figure 5), and angiogenesis (Figure 6). The inflammatory response was not characterized. It was concerned that proinflammatory factors, interleukin (IL)-6, and IL-8, were determined in proteomic analysis of SF, while the anti-inflammatory factor, IL-10, was not (Supplementary Material). Thus, it should be further investigated whether the presence of these proinflammatory factors interrupts the wound healing process (Balaji et al., 2015; Bai et al., 2020). Although the protein levels of TGF-β1 and VEGF were about the same between SF and SC, overall wound healing capability seemed to be much enhanced by SC. We thought that both serum and secreted proteins of SC provide a more favorable environment for wound regeneration than SF; however, it is necessary to confirm how the composition of proteins other than TGF-β1 and VEGF is composed through proteomic analysis of SC.

Recently, as stem cell secretome-based cosmetics have attracted attention, their transdermal delivery efficiency has been disputed. Thus, we estimated the skin permeation of the secretome (Figure 8). In general, it is known that macromolecular drugs, such as peptides and proteins, are impossible to pass through the stratum corneum and can be transported through the follicular route (Bos and Meinardi, 2000). However, due to its uneven distribution depending on the regions, follicular delivery of macromolecules is still ambiguous (Otberg et al., 2004). Likewise, we could observe the transdermal delivery of secretome using macroscopic NIR images and found that the secretome was mainly distributed in the hair follicles and barely transported into the skin surface. There are many types of delivery enhancers to increase the delivery efficacy of macromolecules into the skin (An et al., 2020). It was evidently observed that the secretome was dispersed into the skin besides the follicles when using the non-invasive dermal roller and iontophoresis. Considered that the NIR dye-conjugated secretome proteins were filtrated through 7 kDa MWCO membrane, that is, the molecular weight of the transported proteins was over 7 kDa, it was anticipated that the more active ingredients of pristine secretome actually have an effect on the skin functions when delivered using penetration enhancers.

## Conclusion

In this study, we demonstrated the harvesting and harnessing of the therapeutic potentials of ADSCs in the form of the secretome. Remarkably, Remarkably, we have firstly reported that the maturation process could achieve efficient extraction of secreted factors (i.e., extractomes) from the ADSCs that contains a higher level of secreted molecules than conventional methods. Furthermore, we confirmed that the ADSC secretome acted synergistically to restore skin defect by facilitating tissue regeneration and preventing scar formation without any toxicity by comparing the SC and SF. Consequently, our study implies that ADSC secretome can be effectively produced *via* maturation process, and particularly, SF would be safely utilized to restore damaged tissue architecture in clinical cases.

## Data Availability Statement

The mass spectrometry proteomics data have been deposited to the ProteomeXchange Consortium via the PRIDE (Perez-Riverol et al., 2019) partner repository with the dataset identifier PXD026436.

## Ethics Statement

The animal study was reviewed and approved by Institutional Animal Care and Use Committe (IACUC) at Seoul National University.

## Author Contributions

Y-HA: conceptualization, methodology, validation, writing–original draft, and formal analysis. DHK, S-DK: project administration and supervision. EL: methodology and validation of ADSC secretome. DL and DWK: methodology, validation, and formal analysis of proteomics. MP and JHK: assisted *in vivo* tests and data analysis. JWK, HH, YS, J-YC, and J-UP: writing–review and editing. NH: conceptualization, writing–review and editing, supervision, and project administration. All authors contributed to the article and approved it for publication.

## Conflict of Interest

DHK, EL, JWK, and S-DK were employed by the company, Senior Science & Life, Inc. The remaining authors declare that the research was conducted in the absence of any commercial or financial relationships that could be construed as a potential conflict of interest.

## Footnotes

1 https://www.uniprot.org/
