# Supplementary material for: High-Efficient Production of Adipose-Derived Stem Cell (ADSC) Secretome Through Maturation Process and Its Non-scarring Wound Healing Applications
Source: Front Bioeng Biotechnol. 2021 Jun 16;9:681501. doi: 10.3389/fbioe.2021.681501 (PMC8242583; doi:10.3389/fbioe.2021.681501)
Supplement: Supplementary file 1 [file Data_Sheet_1.PDF]

LFQ intensity Set1 (log2)	N: Intensity	T: Protein names	T: Gene names
42.4454	2.4984E+12	Fibronectin;Anastellin;Ugl-Y1;Ugl-Y2;Ugl-Y3	FN1
39.2763	2.6881E+11	Collagen alpha-1(I) chain	COL1A1
39.156	2.4881E+11	Collagen alpha-3(VI) chain	COL6A3
38.7662	2.0964E+11	72 kDa type IV collagenase;PEX	MMP2
38.7611	2.023E+11	Metalloproteinase inhibitor 1	TIMP1
38.6576	1.719E+11	Pigment epithelium-derived factor	SERPINF1
38.6397	1.6863E+11	Collagen alpha-2(I) chain	COL1A2
38.2656	1.3975E+11	Vimentin	VIM
38.1712	1.5213E+11	Collagen alpha-1(VI) chain	COL6A1
38.0829	1.2829E+11	Transforming growth factor-beta-induced protein ig-h3	TGFBI
37.9915	1.1277E+11	Lumican	LUM
37.7913	1.0266E+11	Biglycan	BGN
37.6807	92058000000	Versican core protein	VCAN
37.4419	72929000000	Laminin subunit gamma-1	LAMC1
37.2672	68713000000	Laminin subunit beta-1	LAMB1
37.1422	63274000000	Collagen alpha-1(XII) chain	COL12A1
37.0803	72334000000	Plasminogen activator inhibitor 1	SERPINE1
36.9475	57060000000	Galectin-3-binding protein	LGALS3BP
36.9235	55327000000	Thrombospondin-1	THBS1
36.9035	50891000000	Collagen alpha-1(III) chain	COL3A1
36.8591	55846000000	Laminin subunit alpha-4	LAMA4
36.8334	53501000000	Follistatin-related protein 1	FSTL1
36.6884	63245000000	SPARC	SPARC
36.3934	38433000000	Collagen alpha-1(V) chain	COL5A1
36.3792	36124000000	Collagen alpha-2(VI) chain	COL6A2
36.3367	39830000000	Nucleobindin-1	NUCB1
36.3057	32496000000	Decorin	DCN
36.2214	31774000000	Basement membrane-specific heparan sulfate proteoglycan core protein;Endorepellin;LG3 peptide	HSPG2
36.164	31238000000	Complement C1s subcomponent;Complement C1s subcomponent heavy chain;Complement C1s subcomponent light chain	C1S
36.1577	33597000000	Procollagen C-endopeptidase enhancer 1	PCOLCE
36.1508	31512000000	Thrombospondin-2	THBS2
36.0857	29053000000	Sulfhydryl oxidase 1	QSOX1
36.0747	29905000000	Complement C1r subcomponent;Complement C1r subcomponent heavy chain;Complement C1r subcomponent light chain	C1R
36.0209	29399000000	Latent-transforming growth factor beta-binding protein 2	LTBP2
35.7769	24625000000	Actin, cytoplasmic 1;Actin, cytoplasmic 1, N-terminally processed;Actin, cytoplasmic 2;Actin, cytoplasmic 2, N-terminally processed	ACTB;ACTG1
35.7681	24773000000	Cartilage oligomeric matrix protein	COMP
35.6817	22661000000	Laminin subunit alpha-2	LAMA2
35.6414	21268000000	Latent-transforming growth factor beta-binding protein 1	LTBP1

35.6152	20437000000	Extracellular matrix protein 1	ECM1
35.5846	21497000000	Fibrillin-1	FBN1
35.4015	23686000000	Pentraxin-related protein PTX3	PTX3
35.3975	26916000000	Immunoglobulin superfamily containing leucine-rich repeat protein	ISLR
35.2753	24453000000	Fibulin-1	FBLN1
35.1914	16966000000	Alpha-actinin-1	ACTN1
35.1587	17659000000	Interstitial collagenase;22 kDa interstitial collagenase;27 kDa interstitial collagenase	MMP1
35.0772	15742000000	Chitinase-3-like protein 1	CHI3L1
35.0568	16920000000	Beta-2-microglobulin;Beta-2-microglobulin form pI 5.3	B2M
35.032	15609000000	Insulin-like growth factor-binding protein 7	IGFBP7
34.9375	12400000000	Cadherin-11	CDH11
34.9374	13329000000	Metalloproteinase inhibitor 2	TIMP2
34.8753	11917000000	Integrin beta-like protein 1	ITGBL1
34.8704	13354000000	Laminin subunit alpha-1	LAMA1
34.8138	13073000000	Procollagen-lysine,2-oxoglutarate 5-dioxygenase 1	PLOD1
34.8101	11921000000	Insulin-like growth factor-binding protein 4	IGFBP4
34.7782	11961000000	Triosephosphate isomerase	TPI1
34.6278	10025000000	Titin	TTN
34.6124	10683000000	Nucleobindin-2;Nesfatin-1	NUCB2
34.5963	10205000000	Collagen triple helix repeat-containing protein 1	CTHRC1
34.5961	10154000000	Prosaposin;Saposin-A;Saposin-B-Val;Saposin-B;Saposin-C;Saposin-D	PSAP
34.5272	10491000000	Filamin-A	FLNA
34.5254	10078000000	Nidogen-1	NID1
34.5235	10128000000	Complement C3;Complement C3 beta chain;C3-beta-c;Complement C3 alpha chain;C3a anaphylatoxin;Acylation stimulating protein;Compleme	C3
34.4756	10120000000	Collagen alpha-2(V) chain	COL5A2
34.418	9160600000	Semaphorin-7A	SEMA7A
34.4076	8781100000	14-3-3 protein epsilon	YWHAE
34.3996	9582900000	Olfactomedin-like protein 3	OLFML3
34.2852	8449900000	Protein disulfide-isomerase A3	PDIA3
34.2798	8570900000	78 kDa glucose-regulated protein	HSPA5
34.2753	8675200000	Agrin;Agrin N-terminal 110 kDa subunit;Agrin C-terminal 110 kDa subunit;Agrin C-terminal 90 kDa fragment;Agrin C-terminal 22 kDa fragment	AGRN
34.1266	7549300000	Stromelysin-1	MMP3
34.0753	7180400000	Complement factor H	CFH
34.0374	7258000000	Target of Nesh-SH3	ABI3BP
33.9851	6844100000	Collagen alpha-1(XI) chain	COL11A1
33.86	6280400000	Sushi repeat-containing protein SRPX	SRPX
33.797	5822500000	Cathepsin B;Cathepsin B light chain;Cathepsin B heavy chain	CTSB
33.7547	5928900000	Clusterin;Clusterin beta chain;Clusterin alpha chain	CLU
33.7113	5953200000	Inhibin beta A chain	INHBA
33.6885	5741300000	Prelamin-A/C;Lamin-A/C	LMNA
33.6767	5957800000	C-type lectin domain family 11 member A	CLEC11A

33.6696	5970300000	Peroxidasin homolog	PXDN
33.6533	5681900000	EGF-containing fibulin-like extracellular matrix protein 1	EFEMP1
33.6444	5153200000	Cathepsin D;Cathepsin D light chain;Cathepsin D heavy chain	CTSD
33.6045	5542500000	Peptidyl-prolyl cis-trans isomerase B	PPIB
33.5833	5011800000	Tenascin	TNC
33.5293	5114400000	Matrix-remodeling-associated protein 5	MXRA5
33.5208	5053600000	Cadherin-2	CDH2
33.5012	4866700000	EMILIN-1	EMILIN1
33.495	4721200000	Spondin-2	SPON2
33.4092	4846900000	CD109 antigen	CD109
33.3914	4537100000	Hemicentin-1	HMCN1
33.381	4433500000	Tropomyosin alpha-4 chain	TPM4
33.3691	4264800000	Annexin A2;Putative annexin A2-like protein	ANXA2;ANXA2P2
33.3548	4572100000	Prostaglandin-H2 D-isomerase	PTGDS
33.2932	6116000000	Cystatin-C	CST3
33.2906	5272600000	Protein-lysine 6-oxidase	LOX
33.2818	4790700000	Adipocyte enhancer-binding protein 1	AEBP1
33.2469	6081300000	Insulin-like growth factor-binding protein 6	IGFBP6
33.2292	4745000000	Phospholipid transfer protein	PLTP
33.1832	4055100000	Calsyntenin-1;Soluble Alc-alpha;CTF1-alpha	CLSTN1
33.1086	4301300000	L-lactate dehydrogenase A chain	LDHA
33.0578	4377500000	Protein disulfide-isomerase	P4HB
33.0245	4285000000	Serine protease HTRA1	HTRA1
33.0202	4459100000	Soluble scavenger receptor cysteine-rich domain-containing protein SSC5D	SSC5D
32.9952	5067600000	45 kDa calcium-binding protein	SDF4
32.975	4210100000	Moesin	MSN
32.9707	3346900000		
32.9343	3327800000	Laminin subunit beta-2	LAMB2
32.9189	3165200000	Transgelin	TAGLN
32.9051	3487600000	Galectin-1	LGALS1
32.8904	3490600000	Prolow-density lipoprotein receptor-related protein 1;Low-density lipoprotein receptor-related protein 1 85 kDa subunit;Low-density lipoprotei	LRP1
32.8407	3186600000	Epididymal secretory protein E1	NPC2
32.8279	3147200000	14-3-3 protein zeta/delta	YWHAZ
32.7101	3470500000	Collagen alpha-1(XV) chain;Restin;Restin-2;Restin-3;Restin-4	COL15A1
32.6714	2662700000	Haptoglobin;Haptoglobin alpha chain;Haptoglobin beta chain;Haptoglobin-related protein	HP;HPR
32.6619	3021700000	Testican-1	SPOCK1
32.6222	2658800000	Cadherin-13	CDH13
32.6125	4016500000	Dickkopf-related protein 3	DKK3
32.6079	2557000000	Rab GDP dissociation inhibitor beta	GDI2
32.5821	2911200000	Vasorin	VASN
32.5547	2807800000	L-lactate dehydrogenase B chain	LDHB

32.5473	2943000000	Macrophage colony-stimulating factor 1;Processed macrophage colony-stimulating factor 1	CSF1
32.5452	2604400000	Alpha-actinin-4	ACTN4
32.4945	2425500000	Golgi membrane protein 1	GOLM1
32.4871	2513900000	Nidogen-2	NID2
32.4802	2604600000	Beta-hexosaminidase subunit alpha	HEXA
32.4594	2852400000	Plasma protease C1 inhibitor	SERPING1
32.429	2375700000	Plectin	PLEC
32.4118	2374500000	Aminopeptidase N	ANPEP
32.3951	2444900000	Gelsolin	GSN
32.373	2814600000	Beta-hexosaminidase subunit beta;Beta-hexosaminidase subunit beta chain B;Beta-hexosaminidase subunit beta chain A	HEXB
32.3613	2310400000	Insulin-like growth factor-binding protein 5	IGFBP5
32.334	2719900000	Phosphoglycerate mutase 1	PGAM1
32.3193	2017200000	Protein disulfide-isomerase A6	PDIA6
32.3007	2001400000	EGF-containing fibulin-like extracellular matrix protein 2	EFEMP2
32.2715	1949400000	Vinculin	VCL
32.2677	2632600000	Microfibril-associated glycoprotein 4	MFAP4
32.2673	2446000000	Serpin H1	SERPINH1
32.2508	2150800000	Myosin-9	MYH9
32.2247	2183400000	Glucose-6-phosphate isomerase	GPI
32.2236	2254400000	Fructose-bisphosphate aldolase A	ALDOA
32.2167	1902600000	Glia-derived nexin	SERPINE2
32.1993	2089500000	Procollagen-lysine,2-oxoglutarate 5-dioxygenase 3	PLOD3
32.1582	1823800000	Cathepsin Z	CTSZ
32.1541	1917200000	Fibulin-5	FBLN5
32.1037	1713600000	Insulin-like growth factor-binding protein 3	IGFBP3
32.0962	2196100000	Apolipoprotein E	APOE
32.0862	1687100000	Beta-1,4-galactosyltransferase 1;Lactose synthase A protein;N-acetyllactosamine synthase;Beta-N-acetylglucosaminylglycopeptide beta-1,4-gala	B4GALT1
32.0843	1734200000	Complement factor B;Complement factor B Ba fragment;Complement factor B Bb fragment	CFB
32.0448	1779200000	Fanconi anemia group M protein	FANCM
31.9913	1971900000	Inactive tyrosine-protein kinase 7	PTK7
31.9613	1591100000	Desmoplakin	DSP
31.9575	2137000000	Thioredoxin domain-containing protein 5	TXNDC5
31.9533	2556900000	Retinol-binding protein 4;Plasma retinol-binding protein(1-182);Plasma retinol-binding protein(1-181);Plasma retinol-binding protein(1-179);Pla	RBP4
31.9107	1642600000	Exostosin-2	EXT2
31.8997	1488700000	Myristoylated alanine-rich C-kinase substrate	MARCKS
31.8773	1696800000	Cathepsin L1;Cathepsin L1 heavy chain;Cathepsin L1 light chain	CTSL
31.8604	1543800000	Alpha-enolase	ENO1
31.8563	1509700000	Fibulin-2	FBLN2
31.856	1542400000	Peptidyl-prolyl cis-trans isomerase A;Peptidyl-prolyl cis-trans isomerase A, N-terminally processed	PPIA
31.849	1679500000	Exostosin-1	EXT1
31.84	1542600000	Peroxiredoxin-1	PRDX1

31.8015	1597300000	Fibrillin-2	FBN2
31.7419	1431400000	Serglycin	SRGN
31.7385	1546100000	Malate dehydrogenase, mitochondrial	MDH2
31.7083	1590700000	Neuropilin-1	NRP1
31.7038	1547600000	Peptidyl-glycine alpha-amidating monooxygenase;Peptidylglycine alpha-hydroxylating monooxygenase;Peptidyl-alpha-hydroxyglycine alpha-am	PAM
31.6874	1516500000	Stanniocalcin-2	STC2
31.6762	1442700000	CD81 antigen	CD81
31.6658	1585600000	Reticulocalbin-3	RCN3
31.6397	1335500000	SH3 domain-binding glutamic acid-rich-like protein 3	SH3BGRL3
31.6389	1633400000	Dystroglycan;Alpha-dystroglycan;Beta-dystroglycan	DAG1
31.6274	1383500000	Endoplasmin	HSP90B1
31.6127	1327800000	Calreticulin	CALR
31.5694	1388000000	V-type proton ATPase subunit S1	ATP6AP1
31.5559	2884100000	Retinoic acid receptor responder protein 2	RARRES2
31.5361	1271400000	Thioredoxin	TXN
31.5315	1203800000	Filamin-C	FLNC
31.5097	1773100000	Ectonucleotide pyrophosphatase/phosphodiesterase family member 2	ENPP2
31.5055	1270400000	C-type mannose receptor 2	MRC2
31.4974	1433200000	Mannosyl-oligosaccharide 1,2-alpha-mannosidase IA	MAN1A1
31.4935	1233700000	Transgelin-2	TAGLN2
31.475	1110900000	14-3-3 protein beta/alpha;14-3-3 protein beta/alpha, N-terminally processed	YWHAB
31.4719	1238900000	Collagen alpha-1(VII) chain	COL7A1
31.461	1153600000	Stanniocalcin-1	STC1
31.4397	1154000000	Beta-mannosidase	MANBA
31.4396	1163000000	Attractin	ATRN
31.4355	1267500000	Granulins;Acrogranin;Paragranulin;Granulin-1;Granulin-2;Granulin-3;Granulin-4;Granulin-5;Granulin-6;Granulin-7	GRN
31.4275	1117700000	Apolipoprotein D	APOD
31.4011	1329500000	Carboxypeptidase E	CPE
31.3837	1054700000	Dermatopontin	DPT
31.3635	1221500000	Calumenin	CALU
31.3465	1217700000	Plastin-3	PLS3
31.3383	1003900000	Brain acid soluble protein 1	BASP1
31.331	1261500000	Nucleoside diphosphate kinase A	NME1
31.3296	1038400000	Heat shock cognate 71 kDa protein	HSPA8
31.3232	1437800000	Retinoic acid receptor responder protein 1	RARRES1
31.315	982800000	Growth/differentiation factor 15	GDF15
31.3072	1306600000	Myosin regulatory light chain 12A;Myosin regulatory light chain 12B;Myosin regulatory light polypeptide 9	MYL12A;MYL12B;
31.301	1007200000	Collagen alpha-1(XVI) chain	COL16A1
31.3005	1197800000	Vitamin K-dependent protein S	PROS1
31.2884	968290000	Histone H2A type 1-J;Histone H2A type 1-H;Histone H2A.J;Histone H2A type 2-C;Histone H2A type 1-C;Histone H2A type 3;Histone H2A type 2	HIST1H2AJ;HIST1
31.2811	1312800000	Ubiquitin-60S ribosomal protein L40;Ubiquitin;60S ribosomal protein L40;Ubiquitin-40S ribosomal protein S27a;Ubiquitin;40S ribosomal protein	UBA52;RPS27A;UB

31.2666	988300000	Out at first protein homolog	OAF
31.2569	1266800000	Glypican-1;Secreted glypican-1	GPC1
31.2257	1112600000	Reticulocalbin-1	RCN1
31.2193	997440000	Heat shock 70 kDa protein 13	HSPA13
31.2091	1051100000	Inactive serine protease PAMR1	PAMR1
31.1996	1062400000	Collagen alpha-1(XVIII) chain;Endostatin	COL18A1
31.1765	949860000	Lysyl oxidase homolog 1	LOXL1
31.1571	910380000	Connective tissue growth factor	CTGF
31.1496	939570000	Glucosidase 2 subunit beta	PRKCSH
31.1072	1088700000	Amyloid beta A4 protein;N-APP;Soluble APP-alpha;Soluble APP-beta;C99;Beta-amyloid protein 42;Beta-amyloid protein 40;C83;P3(42);P3(40);C8	APP
31.0081	936040000	Endosialin	CD248
31.0063	1051600000	ADAMTS-like protein 1	ADAMTSL1
30.9831	837430000	Angiopoietin-related protein 2	ANGPTL2
30.9781	922400000	Lysyl oxidase homolog 2	LOXL2
30.957	848570000	Collagen alpha-2(IV) chain;Canstatin	COL4A2
30.9462	1017500000	Angiopoietin-related protein 4	ANGPTL4
30.933	847350000	Protein FAM3C	FAM3C
30.9318	842880000	Matrix-remodeling-associated protein 8	MXRA8
30.9233	819130000	Collagen alpha-1(XIV) chain	COL14A1
30.8979	771780000	Neurotrimin	NTM
30.8976	767630000	Malate dehydrogenase, cytoplasmic	MDH1
30.8919	847710000	Histone H4	HIST1H4A
30.8757	905520000	Urokinase-type plasminogen activator;Urokinase-type plasminogen activator long chain A;Urokinase-type plasminogen activator short chain A;	PLAU
30.8512	747890000	Protein S100-A11;Protein S100-A11, N-terminally processed	S100A11
30.8403	883560000	Myosin light polypeptide 6	MYL6
30.8222	755200000	Talin-1	TLN1
30.8029	748570000	Ribosome-binding protein 1	RRBP1
30.797	844880000	Bone morphogenetic protein 1	BMP1
30.7475	926320000	Prolyl endopeptidase FAP;Antiplasmin-cleaving enzyme FAP, soluble form	FAP
30.7295	678230000	Perilipin-3	PLIN3
30.7265	704480000	Polypeptide N-acetylgalactosaminyltransferase 2;Polypeptide N-acetylgalactosaminyltransferase 2 soluble form	GALNT2
30.7219	651520000	Phosphoglycerate kinase 1	PGK1
30.7164	833620000	Neuroblast differentiation-associated protein AHNAK	AHNAK
30.6954	693520000	Ubiquitin carboxyl-terminal hydrolase isozyme L1	UCHL1
30.6756	845100000	Interleukin-6	IL6
30.6639	934610000	Extracellular serine/threonine protein kinase FAM20C	FAM20C
30.6576	1253400000	Serum paraoxonase/arylesterase 1	PON1
30.6526	673230000	Thymosin beta-4;Hematopoietic system regulatory peptide	TMSB4X
30.6182	874430000	Glutaminyl-peptide cyclotransferase	QPCT
30.614	742700000	Lysosomal protective protein;Lysosomal protective protein 32 kDa chain;Lysosomal protective protein 20 kDa chain	CTSA
30.5858	668350000	Complement factor I;Complement factor I heavy chain;Complement factor I light chain	CFI

30.5834	904650000	Glypican-6;Secreted glypican-6	GPC6
30.5806	1605700000	Telomerase protein component 1	TEP1
30.5748	606150000	HLA class I histocompatibility antigen, A-66 alpha chain;HLA class I histocompatibility antigen, A-43 alpha chain;HLA class I histocompatibility a	HLA-A
30.5697	689020000	Glutathione S-transferase omega-1	GSTO1
30.4967	587630000	Tropomyosin alpha-3 chain	TPM3
30.4757	641250000	Beta-1,4-glucuronyltransferase 1	B4GAT1
30.4606	594350000	Heat shock protein HSP 90-alpha	HSP90AA1
30.4184	710070000	Pappalysin-1	PAPPA
30.4035	671030000	Ribonuclease 4	RNASE4
30.3336	524140000	N-acetylglucosamine-1-phosphotransferase subunit gamma	GNPTG
30.3267	592020000	Fructose-bisphosphate aldolase C	ALDOC
30.2789	506400000	WNT1-inducible-signaling pathway protein 2	WISP2
30.2664	475120000	Olfactomedin-like protein 2B	OLFML2B
30.2615	480580000	Histone-lysine N-methyltransferase SETD2	SETD2
30.259	472670000	Collagen alpha-1(VIII) chain;Vastatin	COL8A1
30.2582	578500000	Collagen alpha-1(IV) chain;Arresten	COL4A1
30.2382	518920000	Annexin A1	ANXA1
30.2173	612190000	Lactadherin;Lactadherin short form;Medin	MFGE8
30.2	455900000	Ribonuclease T2	RNASET2
30.1919	508080000	Cytoskeleton-associated protein 4	CKAP4
30.1878	476990000	Coactosin-like protein	COTL1
30.1853	538430000	Disintegrin and metalloproteinase domain-containing protein 9	ADAM9
30.1721	503770000	Vascular cell adhesion protein 1	VCAM1
30.1677	559570000	Thrombospondin-3	THBS3
30.167	452960000	Profilin-1	PFN1
30.167	844880000	Microfibrillar-associated protein 2	MFAP2
30.1559	505980000	Coiled-coil domain-containing protein 80	CCDC80
30.1411	458030000	Insulin-like growth factor-binding protein 2	IGFBP2
30.1334	456530000	Legumain	LGMN
30.1299	454340000	Complement component C8 beta chain	C8B
30.102	449350000	Filamin-B	FLNB
30.0953	479800000	Transketolase	TKT
30.0897	468940000	Prostaglandin F2 receptor negative regulator	PTGFRN
30.078	449110000	Isocitrate dehydrogenase [NADP] cytoplasmic	IDH1
30.0596	583170000	Spectrin alpha chain, non-erythrocytic 1	SPTAN1
30.0484	621550000	Vesicular integral-membrane protein VIP36	LMAN2
30.0366	590570000	Chitinase domain-containing protein 1	CHID1
30.0339	452230000	Tumor necrosis factor-inducible gene 6 protein	TNFAIP6
30.0233	563590000	Heterogeneous nuclear ribonucleoproteins A2/B1	HNRNPA2B1
30.0103	456160000	Growth arrest-specific protein 6	GAS6
30.0037	539550000	Polypeptide N-acetylgalactosaminyltransferase 10	GALNT10

29.9396	496950000	Prolyl 3-hydroxylase 1	LEPRE1
29.9246	413700000	Alpha-1-antichymotrypsin;Alpha-1-antichymotrypsin His-Pro-less	SERPINA3
29.9081	402250000	Pyruvate kinase PKM	PKM
29.8987	397650000	Peptidyl-prolyl cis-trans isomerase FKBP10	FKBP10
29.8939	385280000	Interleukin-8;MDNCF-a;Interleukin-8;IL-8(5-77);IL-8(6-77);IL-8(7-77);IL-8(8-77);IL-8(9-77)	CXCL8
29.883	401140000	Endoplasmic reticulum aminopeptidase 1	ERAP1
29.8765	464170000	Neogenin	NEO1
29.8557	359060000	Mitogen-activated protein kinase kinase kinase 19	MAP3K19
29.8556	376420000	DnaJ homolog subfamily C member 3	DNAJC3
29.8525	402520000	Sushi, von Willebrand factor type A, EGF and pentraxin domain-containing protein 1	SVEP1
29.8397	450220000	Multiple inositol polyphosphate phosphatase 1	MINPP1
29.8324	357790000	Protein phosphatase 1 regulatory subunit 14B	PPP1R14B
29.8244	429720000	Sialate O-acetylesterase	SIAE
29.8196	419040000	Glutathione S-transferase P	GSTP1
29.8171	379000000	Cofilin-1	CFL1
29.7869	369770000	Sushi repeat-containing protein SRPX2	SRPX2
**29.7679**	**399520000**	**Vascular endothelial growth factor C**	**VEGFC**
29.7543	336420000	Glyceraldehyde-3-phosphate dehydrogenase	GAPDH
29.7224	393710000	Polypeptide N-acetylgalactosaminyltransferase 5	GALNT5
29.6733	319190000	UTP--glucose-1-phosphate uridylyltransferase	UGP2
29.6581	358940000	Ras GTPase-activating-like protein IQGAP1	IQGAP1
29.6553	362340000	Ganglioside GM2 activator;Ganglioside GM2 activator isoform short	GM2A
29.6231	331040000	Caldesmon	CALD1
29.622	387700000	Collectin-12	COLEC12
29.6202	472060000	Di-N-acetylchitobiase	CTBS
29.6171	314420000	Latent-transforming growth factor beta-binding protein 3	LTBP3
29.6112	371880000	Plasma alpha-L-fucosidase	FUCA2
29.596	345360000	Plexin-B2	PLXNB2
29.5844	562140000	Protein FAM20A	FAM20A
29.5782	315690000	Anthrax toxin receptor 1	ANTXR1
29.5715	304870000	14-3-3 protein gamma;14-3-3 protein gamma, N-terminally processed	YWHAG
29.5402	305410000	Lamin-B2	LMNB2
29.5197	294040000	40S ribosomal protein S28	RPS28
29.5136	607260000	Complement component C6	C6
29.499	286200000	60S acidic ribosomal protein P2	RPLP2
29.4957	288960000	Putative phospholipase B-like 2;Putative phospholipase B-like 2 32 kDa form;Putative phospholipase B-like 2 45 kDa form	PLBD2
29.4917	322860000	Follistatin-related protein 3	FSTL3
29.4786	328020000	Lysyl oxidase homolog 3	LOXL3
29.4781	305000000	Soluble calcium-activated nucleotidase 1	CANT1
29.4773	337770000	Cation-independent mannose-6-phosphate receptor	IGF2R
29.468	280220000	Spondin-1	SPON1

29.468	368110000	Alpha-mannosidase 2	MAN2A1
29.4636	359990000	Xylosyltransferase 1	XYLT1
29.4459	433420000	Tripeptidyl-peptidase 1	TPP1
29.4424	277710000	Fibronectin type III domain-containing protein 1	FNDC1
29.4368	393360000	Podocan	PODN
29.4132	534580000	Microfibrillar-associated protein 5	MFAP5
29.4071	334290000	Neuropilin-2	NRP2
29.4004	260690000	Tropomyosin alpha-1 chain	TPM1
29.3749	267310000	Transitional endoplasmic reticulum ATPase	VCP
29.3711	256460000	Syntenin-1	SDCBP
29.3623	364230000	Growth-regulated alpha protein;GRO-alpha(4-73);GRO-alpha(5-73);GRO-alpha(6-73)	CXCL1
29.3545	413330000	Complement C4-B;Complement C4 beta chain;Complement C4-B alpha chain;C4a anaphylatoxin;C4b-B;C4d-B;Complement C4 gamma chain;Co	C4B;C4A
29.3511	262220000	Renin receptor	ATP6AP2
29.3341	273230000	Major prion protein	PRNP
29.3176	253580000	Superoxide dismutase [Cu-Zn]	SOD1
29.3013	270020000	Thy-1 membrane glycoprotein	THY1
29.2809	257280000	Rho GDP-dissociation inhibitor 1	ARHGDIA
29.2056	304730000	Exostosin-like 2;Processed exostosin-like 2	EXTL2
29.1972	310440000	Gamma-glutamyl hydrolase	GGH
29.1732	319730000	Acyl-CoA-binding protein	DBI
29.1524	238380000	Syndecan-4	SDC4
29.1512	238270000	Interleukin-1 receptor accessory protein	IL1RAP
29.1495	219060000	Aspartate aminotransferase, mitochondrial	GOT2
29.1469	223650000	Follistatin	FST
29.146	248080000	Actin-related protein 3	ACTR3
29.1435	267190000	Galectin-3	LGALS3
29.1419	224900000	Histone H2B type 3-B;Histone H2B type 2-E;Histone H2B type 1-B;Histone H2B type 1-O;Histone H2B type 1-J;Histone H2B type 1-L;Histone H2	HIST3H2BB;HIST2
29.1103	222410000	Protein CutA	CUTA
29.0963	230380000	Arylsulfatase A;Arylsulfatase A component B;Arylsulfatase A component C	ARSA
29.0808	215250000	Calpain-2 catalytic subunit	CAPN2
29.0578	215460000	Elongation factor 2	EEF2
29.0488	307010000	Endothelial protein C receptor	PROCR
29.0394	214860000	Ubiquitin-conjugating enzyme E2 N;Putative ubiquitin-conjugating enzyme E2 N-like	UBE2N;UBE2NL
29.0295	258210000	Meteorin-like protein	METRNL
29.0289	250750000	Carbonic anhydrase 12	CA12
28.9999	201070000	Glucosylceramidase	GBA
28.9596	246770000	Endoplasmic reticulum resident protein 44	ERP44
28.9578	217410000	Dihydropyrimidinase-related protein 2	DPYSL2
28.9459	216160000	Procollagen-lysine,2-oxoglutarate 5-dioxygenase 2	PLOD2
28.9364	216730000	Proteasome subunit alpha type-5	PSMA5
28.9324	251070000	Disintegrin and metalloproteinase domain-containing protein 10	ADAM10

28.9289	225070000	Peroxiredoxin-6	PRDX6
28.8967	193440000	Endoglin	ENG
28.8943	186110000	Lamin-B1	LMNB1
28.8863	182540000	Matrix metalloproteinase-14	MMP14
28.8794	181670000	Protein deglycase DJ-1	PARK7
28.8485	180880000	Peroxiredoxin-2	PRDX2
28.8465	250080000	Neutral alpha-glucosidase AB	GANAB
28.8296	178270000	Rab GDP dissociation inhibitor alpha	GDI1
28.8229	192250000	Platelet-derived growth factor C;Platelet-derived growth factor C, latent form;Platelet-derived growth factor C, receptor-binding form	PDGFC
28.8137	179110000	Integrin beta-1	ITGB1
**28.8089**	**204930000**	**Vascular endothelial growth factor A**	**VEGFA**
28.7992	171840000	Proteasome subunit beta type-1	PSMB1
28.7925	266970000	Platelet-derived growth factor receptor beta	PDGFRB
28.7864	213780000	Protein S100-A13	S100A13
28.7809	190910000	14-3-3 protein theta	YWHAQ
28.7682	218910000	Biotinidase	BTD
28.7662	167960000	Neuronal growth regulator 1	NEGR1
28.7366	314020000	Leukemia inhibitory factor	LIF
28.7358	188710000	Low-density lipoprotein receptor	LDLR
28.7341	254700000	Aspartate aminotransferase, cytoplasmic	GOT1
28.7286	163640000	Peptidyl-prolyl cis-trans isomerase FKBP1A	FKBP1A
28.6956	193260000	Acid ceramidase;Acid ceramidase subunit alpha;Acid ceramidase subunit beta	ASAH1
**28.6919**	**164010000**	**Transforming growth factor beta-1;Latency-associated peptide**	**TGFB1**
28.6578	160910000	Xaa-Pro dipeptidase	PEPD
28.6403	194290000	Heterogeneous nuclear ribonucleoprotein A1;Heterogeneous nuclear ribonucleoprotein A1, N-terminally processed	HNRNPA1
28.628	271030000	Disintegrin and metalloproteinase domain-containing protein 12	ADAM12
28.6271	187680000	Cathepsin F	CTSF
28.5897	169630000	Protein disulfide-isomerase A4	PDIA4
28.5893	172660000	Hepatocyte growth factor-like protein;Hepatocyte growth factor-like protein alpha chain;Hepatocyte growth factor-like protein beta chain	MST1
28.5705	196380000	Proteasome subunit alpha type-6	PSMA6
28.5663	168630000	Multiple epidermal growth factor-like domains protein 8	MEGF8
28.5595	169710000	Calponin-3	CNN3
28.547	252280000	Cystatin-B	CSTB
28.5431	152490000	Early endosome antigen 1	EEA1
28.5369	181050000	Lysosome-associated membrane glycoprotein 1	LAMP1
28.5358	143160000	Proteasome subunit alpha type-4	PSMA4
28.4693	140780000	Probable E3 ubiquitin-protein ligase HECTD4	HECTD4
28.4646	139890000	Cytochrome c	CYCS
28.4611	187980000	Plexin domain-containing protein 2	PLXDC2
28.4594	144880000	Ribonuclease inhibitor	RNH1
28.4504	141900000	Superoxide dismutase [Mn], mitochondrial	SOD2

28.4425	161210000	A disintegrin and metalloproteinase with thrombospondin motifs 12	ADAMTS12
28.4425	189600000	Epididymis-specific alpha-mannosidase	MAN2B2
28.3789	134160000	Protein OS-9	OS9
28.3634	151090000	UDP-glucose:glycoprotein glucosyltransferase 1	UGGT1
28.354	133960000	Calpain small subunit 1	CAPNS1
28.3407	161130000	Protein FAM198B	FAM198B
28.3051	134610000	Nucleophosmin	NPM1
28.2862	185100000	Receptor-type tyrosine-protein phosphatase kappa	PTPRK
28.2805	119940000	Microtubule-associated protein 4	MAP4
28.2784	128920000	Hypoxia up-regulated protein 1	HYOU1
28.2752	230020000	Elongation factor 1-gamma	EEF1G
28.2551	118790000	Proteasome subunit beta type-4	PSMB4
28.2076	127140000	Laminin subunit alpha-5	LAMA5
28.2068	178130000	Urokinase plasminogen activator surface receptor	PLAUR
28.1818	134190000	Protein S100-A16	S100A16
28.1748	169240000	Apolipoprotein M	APOM
28.1726	120220000	Heterogeneous nuclear ribonucleoprotein K	HNRNPK
28.1698	118240000	Lysosomal Pro-X carboxypeptidase	PRCP
28.1676	124640000	Latent-transforming growth factor beta-binding protein 4	LTBP4
28.1481	297460000	CD44 antigen	CD44
28.1468	109330000	Cartilage-associated protein	CRTAP
28.1347	123020000	Src substrate cortactin	CTTN
28.1325	113500000	Spectrin beta chain, non-erythrocytic 1	SPTBN1
28.0911	105190000	Chloride intracellular channel protein 4	CLIC4
28.0883	104980000	Protein-L-isoaspartate(D-aspartate) O-methyltransferase	PCMT1
28.0767	108390000	Transmembrane glycoprotein NMB	GPNMB
28.0606	102990000	RNA-binding motif protein, X chromosome;RNA-binding motif protein, X chromosome, N-terminally processed	RBMX
28.0584	124380000	Signal peptide, CUB and EGF-like domain-containing protein 3	SCUBE3
28.0453	111110000	Twinfilin-1	TWF1
28.0243	102070000	Fatty acid-binding protein, epidermal	FABP5
28.0172	104230000	Matrix metalloproteinase-19	MMP19
28.0146	101400000	Band 4.1-like protein 3;Band 4.1-like protein 3, N-terminally processed	EPB41L3
27.995	110980000	Complement C1r subcomponent-like protein	C1RL
27.9858	118200000	Nucleotide exchange factor SIL1	SIL1
27.9835	130770000	Fibulin-7	FBLN7
27.9715	211190000	CD59 glycoprotein	CD59
27.9494	127040000	Putative sodium-coupled neutral amino acid transporter 10	SLC38A10
27.9352	126560000	5-nucleotidase	NT5E
27.9333	135360000	Alpha-N-acetylglucosaminidase;Alpha-N-acetylglucosaminidase 82 kDa form;Alpha-N-acetylglucosaminidase 77 kDa form	NAGLU
27.9333	94288000	Phosphoserine aminotransferase	PSAT1
27.933	94907000	Protein NDNF	NDNF

27.9021	92272000	Phosphoinositide-3-kinase-interacting protein 1	PIK3IP1
27.8999	133690000	Eukaryotic translation initiation factor 5A-1;Eukaryotic translation initiation factor 5A-2;Eukaryotic translation initiation factor 5A-1-like	EIF5A;EIF5A2;EIF5
27.891	105650000	Adenylyl cyclase-associated protein 1	CAP1
27.8865	91282000	Poliovirus receptor	PVR
27.8864	91273000	BTB/POZ domain-containing protein KCTD12	KCTD12
27.8859	91246000	Alpha-L-iduronidase	IDUA
27.8516	94048000	Interleukin enhancer-binding factor 3	ILF3
27.8469	92802000	Mesencephalic astrocyte-derived neurotrophic factor	MANF
27.8394	88350000	UV excision repair protein RAD23 homolog B	RAD23B
27.8221	87297000	Heat shock protein beta-1	HSPB1
27.8187	150900000	4F2 cell-surface antigen heavy chain	SLC3A2
27.8173	94484000	Quinone oxidoreductase PIG3	TP53I3
27.8095	108510000	Fibrous sheath-interacting protein 2	FSIP2
27.8081	94808000	Calpain-1 catalytic subunit	CAPN1
27.7944	122610000	Proteasome activator complex subunit 1	PSME1
27.7874	90763000	Carbohydrate sulfotransferase 14	CHST14
27.7798	95361000	N-acetylglucosamine-6-sulfatase	GNS
27.7602	83630000	Stress-70 protein, mitochondrial	HSPA9
27.7591	83563000	LIM and SH3 domain protein 1	LASP1
27.7536	93736000	Tumor protein D54	TPD52L2
27.7494	90850000	Protocadherin Fat 1;Protocadherin Fat 1, nuclear form	FAT1
27.7288	104760000	Alpha-1,3-mannosyl-glycoprotein 2-beta-N-acetylglucosaminyltransferase	MGAT1
27.7273	103430000	Calnexin	CANX
27.7257	204890000	Complement C2;Complement C2b fragment;Complement C2a fragment	C2
27.7252	140350000	Protein CREG1	CREG1
27.721	89517000	Gamma-enolase	ENO2
27.7095	92497000	Protein S100-A4	S100A4
27.6929	79820000	Vesicle-trafficking protein SEC22b	SEC22B
27.6747	78818000	Reversion-inducing cysteine-rich protein with Kazal motifs	RECK
27.6651	84963000	Chloride intracellular channel protein 1	CLIC1
27.6557	93806000	Carboxypeptidase Q	CPQ
27.6537	98617000	Alcohol dehydrogenase [NADP(+)]	AKR1A1
27.6454	93766000	Polypeptide N-acetylgalactosaminyltransferase 1;Polypeptide N-acetylgalactosaminyltransferase 1 soluble form	GALNT1
27.6405	209230000	Twisted gastrulation protein homolog 1	TWSG1
27.6101	97926000	Fibromodulin	FMOD
27.6077	84145000	Transaldolase	TALDO1
27.5768	73646000	Proteasome subunit alpha type-3	PSMA3
27.5492	72253000	GDH/6PGL endoplasmic bifunctional protein;Glucose 1-dehydrogenase;6-phosphogluconolactonase	H6PD
27.5458	113900000	Heterogeneous nuclear ribonucleoprotein D0	HNRNPD
27.5149	74554000	Interleukin enhancer-binding factor 2	ILF2
27.5142	70517000	Glutathione synthetase	GSS

27.5106	85935000	Peptidyl-prolyl cis-trans isomerase FKBP9	FKBP9
27.4986	148810000	10 kDa heat shock protein, mitochondrial	HSPE1
27.4689	68337000	A disintegrin and metalloproteinase with thrombospondin motifs 4	ADAMTS4
27.4473	100110000	Matrix metalloproteinase-9;67 kDa matrix metalloproteinase-9;82 kDa matrix metalloproteinase-9	MMP9
27.4409	79590000	Chondroitin sulfate proteoglycan 4	CSPG4
27.4398	87127000	Tubulin alpha-1C chain;Tubulin alpha-1A chain;Tubulin alpha-1B chain;Tubulin alpha-4A chain;Tubulin alpha-3E chain	TUBA1C;TUBA1A;
27.3913	84145000	Cathepsin O	CTSO
27.384	105100000	Thioredoxin-dependent peroxide reductase, mitochondrial	PRDX3
27.3817	64331000	Synaptic vesicle membrane protein VAT-1 homolog	VAT1
27.3391	73595000	Fumarylacetoacetase	FAH
27.3383	64367000	Elongation factor 1-delta	EEF1D
27.3342	63745000	Glucosamine-6-phosphate isomerase 1	GNPDA1
27.3216	68694000	Proteasome subunit beta type-5	PSMB5
27.3068	107160000	Proteasome subunit alpha type-1	PSMA1
27.3006	72350000	60S ribosomal protein L10a	RPL10A
27.2947	60566000	Serine protease 23	PRSS23
27.2784	69980000	Alpha-1-antitrypsin;Short peptide from AAT	SERPINA1
27.2726	65824000	Serpin B6	SERPINB6
27.2647	62291000	Disintegrin and metalloproteinase domain-containing protein 17	ADAM17
27.2199	57505000	Proteasome subunit beta type-6	PSMB6
27.2085	70165000	Phosphatidylethanolamine-binding protein 1;Hippocampal cholinergic neurostimulating peptide	PEBP1
27.1786	55886000	Pregnancy-specific beta-1-glycoprotein 5	PSG5
27.1765	67524000	Tryptophan--tRNA ligase, cytoplasmic;T1-TrpRS;T2-TrpRS	WARS
27.1752	55754000	DBH-like monooxygenase protein 1	MOXD1
27.166	58821000	N(G),N(G)-dimethylarginine dimethylaminohydrolase 1	DDAH1
27.1624	74278000	Protocadherin-7	PCDH7
27.1539	55817000	A disintegrin and metalloproteinase with thrombospondin motifs 2	ADAMTS2
27.1342	61920000	Leucine-rich repeat-containing protein 15	LRRC15
27.109	53249000	Oligoribonuclease, mitochondrial	REXO2
27.0966	82911000	Ubiquitin-conjugating enzyme E2 L3	UBE2L3
27.0917	71906000	Thrombospondin-4	THBS4
27.0783	55371000	Collagen alpha-3(V) chain	COL5A3
27.0782	52125000	Trans-Golgi network integral membrane protein 2	TGOLN2
27.062	63617000	Plastin-2	LCP1
27.0539	61700000	CD166 antigen	ALCAM
27.0393	50739000	Endoplasmic reticulum resident protein 29	ERP29
27.0283	84371000	Lactoylglutathione lyase	GLO1
27.0173	49973000	NKG2D ligand 2;Retinoic acid early transcript 1L protein;Retinoic acid early transcript 1G protein	ULBP2;RAET1L;RA
27.0153	80605000	Elongation factor 1-beta	EEF1B2
27.0102	97657000	N-acetylgalactosamine-6-sulfatase	GALNS
27.0066	57432000	Serpin B7	SERPINB7

26.9834	48810000	ATP synthase subunit delta, mitochondrial	ATP5D
26.9826	68629000	Glutathione reductase, mitochondrial	GSR
26.9688	59982000	EGF-like repeat and discoidin I-like domain-containing protein 3	EDIL3
26.9185	53374000	Sushi, nidogen and EGF-like domain-containing protein 1	SNED1
26.9082	53757000	Metalloproteinase inhibitor 3	TIMP3
26.8989	59277000	Protocadherin gamma-A12	PCDHGA12
26.887	45659000	Cilia- and flagella-associated protein 53	CFAP53
26.8852	45598000	Proteasome subunit alpha type-7	PSMA7
26.8571	44721000	ATP synthase subunit beta, mitochondrial	ATP5B
26.8403	44202000	Cytokine receptor-like factor 1	CRLF1
26.8281	43830000	Discoidin, CUB and LCCL domain-containing protein 1	DCBLD1
26.8162	44862000	Enhancer of rudimentary homolog	ERH
26.8008	43009000	Neudesin	NENF
26.7987	47015000	Prolyl 4-hydroxylase subunit alpha-1	P4HA1
26.7819	61365000	Adenylate kinase 2, mitochondrial;Adenylate kinase 2, mitochondrial, N-terminally processed	AK2
26.7777	42326000	Serine/threonine-protein phosphatase CPPED1	CPPED1
26.7768	48622000	Nucleolin	NCL
26.7727	42176000	Poly(rC)-binding protein 1	PCBP1
26.7525	45112000	Protein transport protein Sec23A;Protein transport protein Sec23B	SEC23A;SEC23B
26.7275	49501000	RGM domain family member B	RGMB
26.7272	40867000	Tissue-type plasminogen activator;Tissue-type plasminogen activator chain A;Tissue-type plasminogen activator chain B	PLAT
26.7265	40849000	Proteasome subunit beta type-2	PSMB2
26.7214	47890000	Thioredoxin reductase 1, cytoplasmic	TXNRD1
26.7148	40516000	Tumor necrosis factor receptor superfamily member 1A;Tumor necrosis factor receptor superfamily member 1A, membrane form;Tumor necros	TNFRSF1A
26.6978	40042000	Hepatoma-derived growth factor	HDGF
26.6966	40010000	Galactocerebrosidase	GALC
26.6926	43366000	Beta-galactosidase	GLB1
26.687	49063000	Programmed cell death 6-interacting protein	PDCD6IP
26.6503	39197000	Actin-related protein 2/3 complex subunit 2	ARPC2
26.6283	44561000	Annexin A6	ANXA6
26.6277	39690000	Ubiquitin-fold modifier 1	UFM1
26.6277	38144000	Nuclear migration protein nudC	NUDC
26.6077	37619000	Secreted frizzled-related protein 4	SFRP4
26.5841	38831000	Polypyrimidine tract-binding protein 1	PTBP1
26.5805	36915000	Major vault protein	MVP
26.5694	46715000	Disintegrin and metalloproteinase domain-containing protein 19	ADAM19
26.563	40168000	Ras suppressor protein 1	RSU1
26.5415	35934000	Dihydropyrimidinase-related protein 3	DPYSL3
26.5269	38751000	Dipeptidyl peptidase 3	DPP3
26.5063	40334000	DNA damage-binding protein 1	DDB1
26.5043	37712000	60S acidic ribosomal protein P0-like;60S acidic ribosomal protein P0	RPLP0P6;RPLP0

26.4713	35395000	Delta-1-pyrroline-5-carboxylate synthase;Glutamate 5-kinase;Gamma-glutamyl phosphate reductase	ALDH18A1
26.4671	34128000	Intercellular adhesion molecule 1	ICAM1
26.4607	33975000	Nicotinamide phosphoribosyltransferase	NAMPT
26.4217	49163000	Stromal cell-derived factor 1;SDF-1-beta(3-72);SDF-1-alpha(3-67)	CXCL12
26.4123	46784000	Lysosomal alpha-glucosidase;76 kDa lysosomal alpha-glucosidase;70 kDa lysosomal alpha-glucosidase	GAA
26.4092	58061000	Protocadherin-18	PCDH18
26.4046	33933000	A-kinase anchor protein 12	AKAP12
26.4022	39715000	Ubiquitin-conjugating enzyme E2 variant 1;Ubiquitin-conjugating enzyme E2 variant 2	UBE2V1;UBE2V2
26.3983	32538000	Protein Wnt-5a	WNT5A
26.3869	32281000	General vesicular transport factor p115	USO1
26.3807	65406000	Kit ligand;Soluble KIT ligand	KITLG
26.3769	49574000	Semaphorin-3C	SEMA3C
26.3651	31798000	Microtubule-associated protein 1B;MAP1B heavy chain;MAP1 light chain LC1	MAP1B
26.3541	37092000	Basigin	BSG
26.3535	40735000	Nodal modulator 1;Nodal modulator 3;Nodal modulator 2	NOMO1;NOMO3;
26.333	40654000	Slit homolog 2 protein;Slit homolog 2 protein N-product;Slit homolog 2 protein C-product	SLIT2
26.3326	38249000	Vacuolar protein sorting-associated protein 26A	VPS26A
26.3006	62773000	Ectonucleotide pyrophosphatase/phosphodiesterase family member 1;Alkaline phosphodiesterase I;Nucleotide pyrophosphatase	ENPP1
26.2628	42958000	Glypican-4;Secreted glypican-4	GPC4
26.2351	29058000	Coronin-1C	CORO1C
26.2276	35720000	Glutathione peroxidase 3	GPX3
26.224	78382000	Dipeptidyl peptidase 1;Dipeptidyl peptidase 1 exclusion domain chain;Dipeptidyl peptidase 1 heavy chain;Dipeptidyl peptidase 1 light chain	CTSC
26.2053	41568000	Lysosomal alpha-mannosidase;Lysosomal alpha-mannosidase A peptide;Lysosomal alpha-mannosidase B peptide;Lysosomal alpha-mannosidas	MAN2B1
26.2023	36730000	F-actin-capping protein subunit alpha-1	CAPZA1
26.1987	31458000	Glyoxalase domain-containing protein 4	GLOD4
26.1831	28029000	Destrin	DSTN
26.1828	33221000	Annexin A5	ANXA5
26.1604	27592000	Endothelial cell-specific molecule 1	ESM1
26.1603	29245000	Tubulin beta chain;Tubulin beta-2B chain;Tubulin beta-2A chain;Tubulin beta-4B chain	TUBB;TUBB2B;TUB
26.1129	32802000	Acid sphingomyelinase-like phosphodiesterase 3a	SMPDL3A
26.1015	37241000	Leucine-rich repeat-containing protein 17	LRRC17
26.0871	47348000	Sialidase-1	NEU1
26.0661	36832000	Growth/differentiation factor 6	GDF6
26.0541	25632000	Integrin alpha-V;Integrin alpha-V heavy chain;Integrin alpha-V light chain	ITGAV
26.0535	33381000	Oncostatin-M-specific receptor subunit beta	OSMR
26.0405	33374000	Proteasome subunit beta type-3	PSMB3
26.0303	62190000	Receptor-type tyrosine-protein phosphatase gamma	PTPRG
26.0291	39020000	Tubulin-specific chaperone A	TBCA
26.0285	33300000	Receptor-type tyrosine-protein phosphatase S	PTPRS
26.0117	24889000	Fin bud initiation factor homolog	FIBIN
26.0071	45950000	Actin-related protein 2/3 complex subunit 1B	ARPC1B

25.9762	24283000	Integrin alpha-5;Integrin alpha-5 heavy chain;Integrin alpha-5 light chain	ITGA5
25.9309	36141000	Protein ERGIC-53	LMAN1
25.913	23244000	Zyxin	ZYX
25.8791	33675000	Catalase	CAT
25.8637	24978000	Golgi apparatus protein 1	GLG1
25.8546	30660000	Fructose-bisphosphate aldolase B	ALDOB
25.8486	25656000	Guanine nucleotide-binding protein G(i) subunit alpha-1;Guanine nucleotide-binding protein G(k) subunit alpha;Guanine nucleotide-binding pr	GNAI1;GNAI3;GN
25.8283	22383000	Heterogeneous nuclear ribonucleoprotein Q;Heterogeneous nuclear ribonucleoprotein R	SYNCRIP;HNRNPR
25.813	41063000	Hyaluronan and proteoglycan link protein 3;Hyaluronan and proteoglycan link protein 4	HAPLN3;HAPLN4
25.8108	21653000	Galactosylgalactosylxylosylprotein 3-beta-glucuronosyltransferase 3	B3GAT3
25.7869	57892000	Proteasome subunit alpha type-2	PSMA2
25.7735	28268000	Integrin beta-5	ITGB5
25.7525	20796000	Tumor necrosis factor receptor superfamily member 6	FAS
25.6917	22868000	Hephaestin	HEPH
25.671	22890000	A disintegrin and metalloproteinase with thrombospondin motifs 7	ADAMTS7
25.648	19343000	Enoyl-CoA delta isomerase 1, mitochondrial	ECI1
25.6243	19028000	Actin-related protein 2/3 complex subunit 3	ARPC3
25.6225	19005000	Neuroendocrine protein 7B2;N-terminal peptide;C-terminal peptide	SCG5
25.6081	38999000	Purine nucleoside phosphorylase	PNP
25.5883	18558000	AP-2 complex subunit beta;AP-1 complex subunit beta-1	AP2B1;AP1B1
25.5808	19287000	Radixin;Ezrin	RDX;EZR
25.5627	26219000	Farnesyl pyrophosphate synthase	FDPS
25.5316	19226000	Growth arrest-specific protein 1	GAS1
25.5213	17717000	Thioredoxin domain-containing protein 17	TXNDC17
25.5169	18846000	Nuclear mitotic apparatus protein 1	NUMA1
25.4245	20690000	Glycosaminoglycan xylosylkinase	FAM20B
25.4071	16369000	Twinfilin-2	TWF2
25.3639	15885000	Phosphoglucomutase-2	PGM2
25.3413	21961000	Proteasome activator complex subunit 2	PSME2
25.3063	15264000	Tissue factor pathway inhibitor	TFPI
25.2957	17363000	Arylsulfatase B	ARSB
25.2811	15000000	Integrin alpha-2	ITGA2
25.2728	16185000	Eukaryotic initiation factor 4A-II;Eukaryotic initiation factor 4A-II, N-terminally processed;Eukaryotic initiation factor 4A-I	EIF4A2;EIF4A1
25.2712	40494000	D-dopachrome decarboxylase;D-dopachrome decarboxylase-like protein	DDT;DDTL
25.2647	17024000	60 kDa heat shock protein, mitochondrial	HSPD1
25.2625	14807000	Transformation/transcription domain-associated protein	TRRAP
25.2578	14759000	Neurogenic locus notch homolog protein 3;Notch 3 extracellular truncation;Notch 3 intracellular domain	NOTCH3
25.2453	14631000	Hepatocyte growth factor receptor	MET
25.2231	14408000	Heat shock 70 kDa protein 4	HSPA4
25.209	14269000	Carboxypeptidase A4	CPA4
25.1344	13549000	Translin	TSN

25.1334	24566000	Heat shock protein HSP 90-beta	HSP90AB1
25.1246	42909000	Double-stranded RNA-specific editase 1	ADARB1
25.0744	29802000	Clathrin heavy chain 1	CLTC
25.059	12859000	Transforming protein RhoA;Rho-related GTP-binding protein RhoC	RHOA;RHOC
25.0481	13700000	Dickkopf-related protein 1	DKK1
25.0129	33855000	Actin-related protein 2	ACTR2
24.9582	16167000	Dipeptidyl peptidase 4;Dipeptidyl peptidase 4 membrane form;Dipeptidyl peptidase 4 soluble form	DPP4
24.9412	32214000	Alpha-1,6-mannosyl-glycoprotein 2-beta-N-acetylglucosaminyltransferase	MGAT2
24.8541	19176000	Solute carrier family 2, facilitated glucose transporter member 1	SLC2A1
24.8283	10959000	40S ribosomal protein S12	RPS12
24.8137	18132000	Protocadherin-16	DCHS1
24.7489	11749000	Alpha-(1,6)-fucosyltransferase	FUT8
24.7093	14575000	Tetraspanin-6	TSPAN6
24.6941	13198000	Heparan sulfate glucosamine 3-O-sulfotransferase 3B1;Heparan sulfate glucosamine 3-O-sulfotransferase 3A1;Heparan sulfate glucosamine 3-O	HS3ST3B1;HS3ST3
24.6561	26437000	Serine/threonine-protein kinase ATR	ATR
24.6298	13843000	Matrix-remodeling-associated protein 7	MXRA7
24.6275	13065000	LIM domain only protein 7	LMO7
24.6022	25469000	1,4-alpha-glucan-branching enzyme	GBE1
24.586	25184000	Probable carboxypeptidase X1	CPXM1
24.5428	10857000	Cellular retinoic acid-binding protein 2	CRABP2
24.5097	12666000	Small nuclear ribonucleoprotein Sm D2	SNRPD2
24.4974	23684000	Extracellular sulfatase Sulf-2	SULF2
24.4521	9176700	Epidermal growth factor receptor	EGFR
24.369	10041000	SH3 domain-binding glutamic acid-rich-like protein	SH3BGRL
24.3414	13919000	UDP-GlcNAc:betaGal beta-1,3-N-acetylglucosaminyltransferase 9	B3GNT9
24.2559	20033000	Membrane-bound transcription factor site-1 protease	MBTPS1
24.1984	7082000	182 kDa tankyrase-1-binding protein	TNKS1BP1
24.1972	11677000	Growth factor receptor-bound protein 2	GRB2
24.1675	18843000	Quinone oxidoreductase	CRYZ
24.1044	17374000	Alkaline phosphatase, tissue-nonspecific isozyme	ALPL
23.7923	10466000	Junction plakoglobin;Catenin beta-1	JUP;CTNNB1
23.6553	13212000	Palmitoyl-protein thioesterase 1	PPT1
23.6074	12780000	MICOS complex subunit MIC60	IMMT
23.591	7472500	Cell growth regulator with EF hand domain protein 1	CGREF1
23.554	6951500	Eukaryotic translation initiation factor 4 gamma 1	EIF4G1
23.5062	11914000	Transferrin receptor protein 1;Transferrin receptor protein 1, serum form	TFRC
23.4634	11566000	Fumarate hydratase, mitochondrial	FH
23.4593	4242600	Aspartyl/asparaginyl beta-hydroxylase	ASPH
23.3613	3964100	V-type proton ATPase catalytic subunit A	ATP6V1A
23.3484	10680000	WD repeat-containing protein 1	WDR1
23.2504	9978800	Alpha-2-macroglobulin receptor-associated protein	LRPAP1

23.2365	3635700	Frizzled-2;Frizzled-7	FZD2;FZD7
22.9918	3068600	Dynactin subunit 1	DCTN1
22.8647	5340800	Netrin-G1	NTNG1
22.8527	7574200	ADP-ribose pyrophosphatase, mitochondrial	NUDT9
22.7694	7149600	26S proteasome non-ATPase regulatory subunit 2	PSMD2
22.5068	5959500	Cadherin-4	CDH4
22.4003	5535400	Sphingomyelin phosphodiesterase	SMPD1
22.3823	2011200	Ras GTPase-activating protein-binding protein 2	G3BP2
21.9435	1483700	Nuclease-sensitive element-binding protein 1	YBX1
20.5949	1583700	Heat shock protein beta-6	HSPB6
